# Effectiveness of Parent-Child Interaction Therapy (PCIT) in the Treatment of Young Children’s Behavior Problems. A Randomized Controlled Study

**DOI:** 10.1371/journal.pone.0159845

**Published:** 2016-09-13

**Authors:** Åse Bjørseth, Lars Wichstrøm

**Affiliations:** 1 Levanger Hospital, Nord-Trøndelag Hospital Trust, Levanger, Nord-Trøndelag, Norway; 2 Department of Psychology, Norwegian University of Science and Technology, Sør-Trøndelag, Trondheim, Norway; University of Queensland, AUSTRALIA

## Abstract

**Objective:**

The aim of the present investigation was to compare the effectiveness of Parent-Child Interaction Therapy (PCIT) with treatment as usual (TAU) in young children who were referred to regular child and adolescent mental health clinics for behavior problems.

**Method:**

Eighty-one Norwegian families with two- to seven-year-old children (52 boys) who had scored ≥ 120 on the Eyberg Child Behavior Inventory (ECBI) were randomly assigned to receive either PCIT or TAU. The families were assessed 6 and 18 months after beginning treatment. Parenting skills were measured using the Dyadic Parent-Child Interaction Coding System (DPICS), and child behavior problems were measured using the ECBI and the Child Behavior Checklist (CBCL).

**Results:**

Linear growth curve analyses revealed that the behavior problems of children receiving PCIT improved more compared with children receiving TAU according to mother reports (ECBI *d* = .64, CBCL *d* = .61, both *p* < .05) but not according to father report. Parents also improved with regard to Do and Don’t skills (*d* = 2.58, *d* = 1.46, respectively, both *p* ≤ .001). At the 6-month assessment, which often occurred before treatment was finished, children who had received PCIT had lower father-rated ECBI and mother-rated CBCL-scores (*p* = .06) compared with those who had received TAU. At the 18-month follow-up, the children who had received PCIT showed fewer behavior problems compared with TAU according to mother (*d* = .37) and father (*d* = .56) reports on the ECBI and mother reports on the CBCL regarding externalizing problems (*d* = .39). Parents receiving PCIT developed more favorable Do Skills (6-month *d* = 1.81; 18-month *d* = 1.91) and Don’t Skills (6-month *d* = 1.46; 18-month *d* = 1.42) according to observer ratings on the DPICS compared with those receiving TAU.

**Conclusion:**

Children receiving PCIT in regular clinical practice exhibited a greater reduction in behavior problems compared with children receiving TAU, and their parents' parenting skills improved to a greater degree compared with those receiving TAU.

**Trial Registration:**

ClinicalTrials.gov NTC01085305

## Introduction

Behavior problems [e.g., symptoms of oppositional defiant disorder (ODD) and conduct disorder (CD)] are a primary reason for the referral of young children to treatment [1], and they are prevalent in the population [2]. These symptoms often manifest during the toddler and early preschool years, and if left untreated, such early emerging behavior problems tend to persist and often have a range of negative long-term outcomes, including dropping out of school and developing antisocial personality disorder [3]. Therefore, to interrupt the dysfunctional and detrimental trajectories these children may follow, early intervention is essential.

A range of parent behavior training (BPT) programs have proven to be efficacious in treating behavior problems in young children [4], and BPT is the treatment of choice for young children [5]. The main essence of BPTs is to teach parents to interact differently, i.e., to use more appropriate and positive parenting practices with their children in order to lessen symptoms of behavioral disorders. Four BPTs have been given the highest possible rating as “well supported by research evidence” by the California Evidence-Based Clearinghouse for Child Welfare (http://www.cebc4cw.org). These interventions are Parent Management Training-Oregon (PMT-O [6]), the Incredible Years parent training (IYPT [7]), Triple-P Positive Parenting Program (Triple-P [8–10]), and Parent-Child Interaction Therapy (PCIT [11, 12]). These programs have been demonstrated to be effective in improving child behavior in a diverse range of families. They have also demonstrated effectiveness in a variety of community settings around the world [13–18].

PCIT differs from the three other interventions in that parents and children meet together in dyadic parent-child sessions where parent coaching is performed in vivo through a wireless in-ear speaker with the child and the parent in a playroom while the therapist works from behind a one-way mirror. Thus, the therapist sees the child throughout therapy and can tailor the treatment according to directly observed parent and child behavior instead of solely relying on parent reports. PCIT was originally developed as an intervention for children with disruptive behavior problems such as ODD or CD, and there is a large body of research to support the efficacy of PCIT for such externalizing problems in young children [11, 12]. PCIT has also shown promising effects on a range of problems beyond externalizing, e.g. depression [19], anxiety [20, 21], and it also shows applicability to very young children [22, 23]. First and foremost, the individually tailored in-vivo coaching was the reason that PCIT was chosen as the parent-training program of choice for the present investigation.

Except for one study from the Netherlands [17], PCIT has not been investigated in Europe. Moreover, given the more permissive and democratic parenting styles suggested to be typical of the Nordic countries [24], parenting programs originating in the U.S.A. may not have the same effect in these countries. It may therefore be of particular importance to investigate the effect of PCIT in a Nordic country such as Norway.

### Efficacy Studies and Routine Care

Efficacy-studies are mostly conducted in research settings, far from routine settings in communities, and few studies have moved PCIT from research settings to clinical practice in real-world conditions. Concerns have been raised about whether evidence-based programs are indeed more effective compared with usual clinical care and if they are applicable in usual care [25]. According to Weisz and colleagues, clinical practitioners might be reluctant to use evidence-based programs because they have mainly been tested on youths with subclinical problems and may not work well among those with more serious and complex problems above the diagnostic threshold, such as who are treated in real-world settings. They further claim that clinical practitioners often find evidence-based treatments too rigidly manualized to permit the individual tailoring of treatment that professionals often attempt in usual care and that these programs are not easily transferred to other ethnic groups or cultures. As a way of bridging the gap between the research and routine care settings, both Weisz and colleagues [26] and Michelson and colleagues [6] have provided criteria on how to transport studies from research settings to more clinically representative environments. First, they propose that the therapy should be delivered to a clinic-referred population actively seeking services. Second, they suggest that it should be conducted in a routine setting as part of a routine service. Third, both recommend that the trial should be conducted as a randomized controlled study (RCT) with usual care as comparison group.

### Previous PCIT Studies

#### Clinic-referred populations

Although most studies on PCIT have not used patients seeking treatment in routine settings, some have. Promising results have been obtained in studies of ethnic minority populations seeking treatment in usual care [27, 28], in treating traumatized children [29], in the prevention of maltreatment [30], and for children with mental retardation [31]. Some of these studies have examined behavioral problems [17, 18, 27, 28, 32, 33].

#### Routine settings as part of routine service

In regards to those PCIT-studies who have studied clinic-referred populations with behavioral problems (above), several studies have not offered PCIT as part of their routine services by clinicians usually working in these clinics [27, 28, 30–35]. However, a few studies have examined PCIT as part of such routine services in clinic-referred populations who have behavioral problems [17, 18].

#### RCT with TAU as a control

Behavioral programs in general have shown better results compared to waitlist controls examined in clinical settings, but most behavioral programs for behavioral problems including comparison groups have applied waitlist controls as opposed to usual care [6]. In regards to PCIT, the intervention has proven to be superior to waitlist controls in reducing disruptive behavior in young children [18, 33, 36]. However, parent training programs for behavior problems, including PCIT, must also demonstrate to be effective when compared to active treatment [37], though to our knowledge only two studies have applied active treatment group comparisons. First, one study reported a favorable effect of PCIT and a culturally modified version of PCIT in a Mexican-American population at the end of treatment [34] using student therapists. The positive effect of regular PCIT versus TAU was not supported at a two-year follow-up [38]. Because modest sample size and attrition may explain the lack of findings supporting PCIT, there is a need to examine the effectiveness of PCIT in regular clinical practice in routine service by everyday clinicians. Second, in a recent effectiveness trial from the Netherlands, PCIT was compared to Family Creative Therapy (FCT). Both treatments are common therapies in this country and they were conducted by clinicians working in clinics serving the community [17]. This study was originally planned as a randomized controlled study; despite being promising, the results were difficult to interpret because the randomization was broken by allowing families allocated to the TAU group to switch to the PCIT group if they so desired. Hence, we also do not know whether those who remained in the TAU group did so because they preferred the TAU treatment. Finally, Chaffin and colleagues [30], found that PCIT in combination with a self-motivation intervention reduced child welfare recidivism more compared with standard service. However, reports to child welfare and child replacements do not necessarily translate well to child behavior problems. Therefore, we do not know whether PCIT is effective for such child problems.

#### Long-term follow-up

The effects of interventions should preferably be lasting beyond the treatment period, at least to some extent; therefore, the effects of programs need to be examined in long-term follow-ups. Children participating in PCIT have been followed over an extended period in several studies, and the results have evidenced that children receiving PCIT to some extent maintain their initial gains [39, 40]. The lack of control groups makes these findings difficult to interpret *vis-a-vis* the effectiveness of PCIT. One prior study has, however, involved a TAU control group using student therapists and carried out a longer-term follow-up; it failed to find a positive effect [38]. In the present study, we used everyday therapists working in regular clinics and examined the effectiveness of the intervention 6 and 18 months after initiating treatment.

### The Present Investigation

To our knowledge no randomized controlled study of PCIT in usual care using a TAU control group has previously been conducted. The purpose of the present study was, therefore, to evaluate the long-term effectiveness of PCIT in young children referred to regular child and adolescent mental health specialty (CAMHS) clinics in Norway using a TAU control group. The study included all of the aforementioned real-world criteria: (i) the interventions were provided by non-specialist therapists, i.e., practicing clinicians, to treatment-seeking youth in a clinic-referred population; (ii) the interventions were conducted in a clinical service setting as part of a routine service; (iii) the participants were randomized to either PCIT or TAU. Taking the previously reported efficaciousness of PCIT and its aforementioned merits into account, we hypothesized that the children referred to the CAMHS clinics for behavior problems who received PCIT would demonstrate a greater reduction in symptoms compared with the children receiving TAU and that parents receiving PCIT would show greater improvement in parenting skills compared to parents receiving TAU.

## Method

### Participants

**Fig 1:**
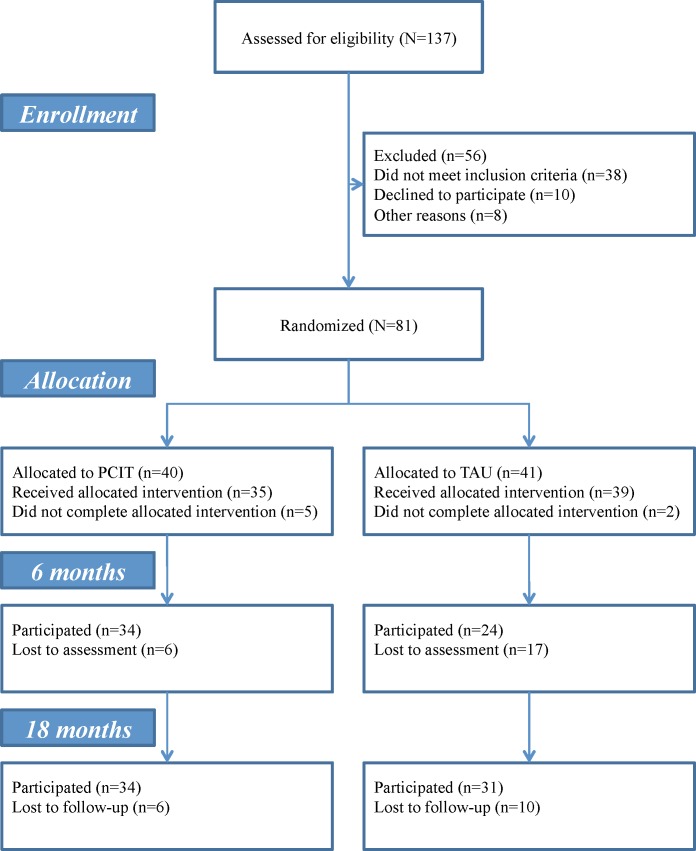
Flow of participants from recruitment through analysis. PCIT = Parent-Child Interaction Therapy, TAU = Treatment as usual.

Children who were consecutively referred to two outpatient CAMHS clinics in Mid-Norway (i.e. Nord-Trøndelag Hospital Trust and St. Olav’s Hospital, Trondheim University Hospital) between November 2006 and December 2010 were included if they were (i) referred for behavior problems, (ii) scored ≥ 120 on the Eyberg Child Behavior Inventory (ECBI [41]), which corresponds to the 90^th^ percentile according to Norwegian norms [42] and (iii) were two to seven years of age. In Norway, three agencies are allowed to refer children to CAMHS; the majority is referred by general practitioners (physicians), some from child-protection services, and in a few cases from other hospitals or clinics. The following exclusion criteria were applied: children with (i) autism spectrum disorders, (ii) mental retardation, and (iii) parents who were not proficient enough in the Norwegian language to complete the ECBI and receive therapy without an interpreter. Fig 1 presents the recruitment and follow-up details. The children ranged in age from 2.1 to 7.7 years. In all but 10 cases (TAU *n* = 7, PCIT *n* = 3), both mothers and fathers participated in the treatment and rated their children. Among the 74 parents with available information on ethnicity, 70 were of Norwegian origin, 2 were from other Western countries, 1 was from South America, and 1 was Asian. There were no differences between PCIT and TAU according to demographic or study variables.

The attrition rate with respect to assessment from pre-test to the 6 and 18-month follow-up were 28.4% and 19.8%, respectively. Fewer participants from the PCIT group compared with those from the TAU-group dropped out of the study at 6 months, χ² (1, n = 81) = 4.17, *p* = 04, but not at 18 months χ² (1, n = 81) = 11 *p* = .74. At one of the clinics there was reduced availability for assessment of TAU families at 6 months and this was a likely reason for the higher dropout rate among TAU families at that time. At 6 months more families with parents who were not cohabitating dropped out (50.0%) compared with cohabitating families (19.3%), χ² (1, n = 69) = 5.03, *p* = .03, which was not the case at 18 months (25.0% versus 17.5%), χ² (1, n = 69) = 0.36, *p* = .55. Moreover, at 18 months there was a tendency for more children with ODD to stay in the study (drop-out rate: 11.6%) than for those without ODD (drop-out rate: 32.3%) χ² (1, n = 74) = 3.55, *p* = .06. Apart from this, attrition was not related to any demographic or study variables.

### Procedure

All families who met the inclusion criteria were asked to participate in the study. Therapists at the participating clinics provided verbal and written information about the study, enrolled the participants and assigned them to interventions. Consenting parents signed a written consent form and completed a diagnostic interview the Preschool Age Psychiatric Assessment (PAPA [43]), and were then assigned to either the PCIT (n = 40) or TAU (n = 41) group. Because earlier studies on PCIT had used wait-list controls, the effect estimations were uncertain. Thus we additionally based our calculations on the effects achieved by the two most-used parenting programs, The Incredible Years [44] and Parent Management Training-Oregon [45]. At the time that the study was planned, in 2006, effect sizes varied considerably, from .20 [46] to 1.05 [47] to 1.59 [48] with regard to parent-reported behavior problems, similar to the methods used in the present study. We therefore took a middle value of *d* = .80 as an expected outcome. The average dropout rate in PCIT studies published at the time the study was planned was 30%, and we expected an additional 15% drop out by the 18-month follow up. Given these considerations we expected to achieve a power of .80 with 84 families initially recruited and equally allocated to the two conditions when using a two-tailed test and p < .05 at the 18-month assessment, calculated by G-power 3.1.9.2, [49] using the expected difference in means between two independent groups. Assignment to either the PCIT group or the TAU group was conducted using a random number generator. The numbers were sent to the clinics in consecutively numbered sealed envelopes, which were opened after the assessments were completed. An individual external to the study and not otherwise involved in the treatment was tasked with administering the group assignment.

PCIT continues until parents master the communication skills at pre-specified levels. Thus, assessing children immediately after treatment ends may introduce a difference between PCIT and TAU due to differences in the treatment progression, which may confound the outcomes. Therefore, families were followed-up at fixed intervals, 6 and 18 months after initiating treatment, as illustrated by the flow chart in Fig 1. The first families were enrolled in the study and started their pre-treatment assessment in December 2006, and the final follow-up assessment was conducted in January 2013. Originally, five clinics within the two trusts had intended to participate, and the data collection was designed to last two years. However, before data collection started, three of the clinics withdrew from the study due to turnover among the PCIT therapists, and this resulted in an extension of the trial period.

The Regional Ethical Committee for Medical Research, Mid-Norway (number 4.2006.789) approved the study according to the protocol the May 16.2006. The trial is registered with Clinical Trials.gov (number NTC01085305); the authors first became aware of the need to register the study there in 2010. Trial study protocol in Norwegian and English will be found as S1 Text, S2 Text, respectively. The protocol was not changed following the enrollment of families into the study and the approval by the ethics committee. A CONSORT Checklist is also provided as S3 Text. According to the author’s Data Availability Statement, the data are protected for the sake of the anonymity of the participants as per the instruction of the Ethical board, the Regional Ethical Committee for Medical Research, Mid-Norway. This board may grant access to the data upon request (rek-midt@medisin.ntnu.no).

The PAPA was used as a diagnostic assessment in the research part of the study. However, the diagnostic manual used by CAMHS clinics in Norway is the ICD-10 [50], which was used by the CAMHS clinics to diagnose attention deficit hyperactivity disorder diagnosis (ADHD) in the CAMHS. There is a discrepancy between hyperkinetic disorder as identified by ICD-10 criteria and ADHD as identified by the DSM, whereby the ICD-10 identifies a narrower group of children compared with those identified by the DSM [51, 52]. This explains the discrepancy between the percentages of ADHD diagnoses as represented in Table 1 and the descriptions concerning those who received the ADHD diagnosis in the CAMHS clinics. Children who were eligible for the study but who subsequently received an ICD-10 ADHD diagnosis (n = 18) were offered medication according to regular practice. In this period there was no other treatment besides deciding the type and dosage of the medication. If the child was still under the age of seven when this procedure was completed, he or she was re-invited to the study. The child's parents then completed a new ECBI, and if they scored ≥ 120 on the ECBI, the family was re-enrolled into the study (n = 17). Ten children received medication for ADHD (n = 7 in TAU and n = 3 in PCIT), with no difference between the treatment groups, χ^2^ = 1.96, df = 1, *p* = .16.

**Table 1:** Participant demographics according to study group.

	PCIT		TAU	
	*N*	*M* or percentage	*N*	*M* or percentage
Boys	29	50.9	28	49.1
Age	40	5.7	38	5.9
SES				
Unskilled workers	3	8.3	0	0
Skilled workers	18	50.0	22	68.8
Lower professionals	11	30.6	6	18.8
Higher professionals and leaders	4	11.1	4	12.6
Marital status of parental figures				
Married	9	26.5	12	33.3
Cohabitating > 6 months	18	52.9	17	47.2
Cohabitating < 6 months	1	2.9	4	11.1
Separated or divorced	6	17.7	1	2.8
Never lived together	0	0	2	5.6
Informant parent's age	37	34.9	34	36.3
In contact with child protective services	13	32.5	19	46.34
Diagnoses				
ODD	20	58.8	22	61.1
CD	7	20.6	9	25
ADHD	11	32.4	14	38.9
Anxiety	15	44.1	15	41.7
Depressive disorders	6	17.6	4	11.1
Any behavioral disorder	26	76.5	26	72.2
Any emotional disorder	17	50	18	50
Any diagnosis	28	82.4	27	75

Note: SES = Socio-economic status, ODD = Oppositional Defiant Disorder, CD = Conduct Disorder, ADHD = Attention Deficit Disorder.

### Intervention

PCIT consists of two phases: Child-Directed Interaction (CDI) and Parent-Directed Interaction (PDI) [53]. The therapist communicates with the parents from behind a one-way mirror through a wireless in-ear speaker while the parent plays with the child. The aim of the CDI is for parents to learn more appropriate and positive parenting practices and to establish a warm and rewarding relationship, thereby improving the parents’ ability to set limits and consistently follow through. PCIT was conducted as weekly sessions, and both parents were encouraged to attend therapy. When both parents attended, a session was first conducted with one of the parents, and a second session with the other parent immediately followed. The therapy was conducted using the Norwegian translation of the PCIT protocol [54]. The PCIT protocol was revised in 2011; however, to avoid introducing uncontrolled variance into the study and thereby potentially jeopardizing clear interpretation of results, we decided to continue with the 1999 protocol throughout the trial. Due to common practice and the facilities at the clinics, we deviated from the PCIT protocol at one point; instead of the backup procedure in the PDI described in the protocol (i.e., the backup-room), we used one of two alternative backup procedures described by McNeil & Hembree-Kigin [55, 56] (viz. the "swoop-and-go" and "the two-chair procedure"), dependent on the child's age and parental preferences.

Two therapists were present in 45% of the sessions. Based on integrity checklists [54], the co-therapist monitored the procedural fidelity of the other therapist and provided feedback during the sessions. There were no systematic fidelity checks for the cases in which there was no second therapist available. The practitioners used PCIT coding sheets during each session to indicate mastery of the CDI and PDI behaviors. According to the manual, parents were considered "completers" when they mastered both the CDI and PDI in a five-minute period prior to each treatment session according to the established criteria on the DPICS and when also obtaining an ECBI score ≤ 120. On average, families received 21.14 sessions *(SD* = 12.04) of PCIT. TAU implies that the therapists were allowed to determine the type and duration of treatment according to the routine services provided at the CAMHS clinic. There were no measured fidelity checks on the regular services in the TAU group. The length of therapist's experience did not differ between PCIT and TAU, *M*_*PCIT*_ = 8.24 years, *SD* = 5.20; *M*_*TAU*_ = 8.22 years, *SD* = 6.84; t (67.23) = -.02, *p* = .99 (two-tailed).

#### Characteristics of the providers

The therapists were clinical practitioners at the actual CAMHS clinics. PCIT therapists never offered TAU to participating families, and TAU therapists did not offer PCIT. Six therapists provided PCIT, and all had used the program as a part of their routine service before the trial started. On average, the therapists had 3.87 (*SD* = 1.21) years of experience with PCIT before the trial started. Except for one therapist, all were specialists in child and adolescent mental health and had practiced as licensed child therapists for several years prior to the trial (*M* = 8.24 years, *SD* = 5.20). Two of the PCIT therapists (*n* = 6) were trained by PCIT master-trainers. These two agency in-house trainers trained the other therapists. The training was provided in a group format as a 40-hour workshop. In addition the six therapists received advanced training with a master-trainer. The therapists received individual supervision on their first two PCIT cases, and then continued with monthly group supervision, both of which were conducted by the in-house trainers.

There were a total of 10 therapists providing TAU, none of whom was trained in PCIT. All of the therapists were licensed psychologists or social workers, and seven of the 10 therapists were experienced clinicians having received specialized education in child and adolescent psychiatry. Those TAU clinicians who pursued clinical specialization during the trial continued their regular weekly supervision.

#### Characteristics of the control group

Within the TAU, therapists were allowed to determine the type, duration and frequency of treatment, according to common practice in the respective clinics, while excluding the Incredible Years and PTM-O. This exception was made because it was assumed that including interventions in the TAU group similar to those in the PCIT group could reduce the possible effects of treatment between the groups. The 41 controls received diverse interventions (*M*_types_ = 2.5, *SD* = 1.2, range 0 – 5). Individual therapy with the child (*n* = 32) and parent counseling (*n* = 33) were most often used, followed by play therapy (*n* = 12), family therapy (*n* = 12) or Marte Meo, a video-based parent intervention [57] (*n* = 4). A minority received behavior-oriented treatment in the format of the Incredible Years [58] (*n* = 4). This deviation from the trial study protocol was conducted without the authors’ knowledge, and we were therefore unable to prevent it. The analyses were conducted a second time after excluding these four families, without any changes in the results.

On average, the number of TAU sessions was 18.84 (*SD* = 11.78), which did not differ from that of the PCIT group, *t* (76) = .85, *p* = .40. In 15 of the cases more than one therapist worked with the family.

### Instruments

#### Eyberg Child Behavior Inventory

The ECBI [41] was used to measure behavior problems. Norwegian norms for ECBI are available [42], and indications of construct validity comes from Norwegian treatment studies [59]. Both parents (if present) completed the 36-item ECBI questionnaire, which yields an Intensity Scale (i.e., the frequency of disruptive behaviors) (α = .87) and a Problem Scale (i.e., the number of behaviors that parents report as a problem to them) (α = .86).

#### Child Behavior Checklist

The Child Behavior Checklist (CBCL [60, 61]) was used to measure a wider set of problems and symptoms of externalizing problems were used in the present inquiry. A range of studies indicates that the CBCL is validated for Norwegian children [13, 62–64]. The CBCL 1.5–5 version was used for children aged 2–5 years, whereas the 6–18 version was used for older children. The reliabilities of the CBCL were α = .82 and α = .85 in the 1.5–5 version and α = .91 and α = .93 in the 6–18 version, for mothers and fathers, respectively.

#### Dyadic Parent-Child Interaction Coding System

The Dyadic Parent-Child Interaction Coding System (DPICS [65]) is a behavioral observation system designed to assess the quality of parent-child interactions. It consists of three five-minute standard situations: Child-Led Play (CLP), Parent-Led Play (PLP), and Clean-Up (CU). The observations of parent-child interaction with the DPICS were conducted at the CAMHS clinics and were videotaped for later coding. The coders were blinded to all information concerning the family. Ten percent of the DPICS recordings were re-coded by blinded raters. Arguably, a supportive parent may compensate for the effect of harsh or insensitive behavior by the other parent. When analyzing parents’ behavior toward the child separately, such an effect will not be captured. We therefore created sum-scores of mothers' and fathers’ behaviors. A total of 18 parent and eight child categories were coded from the videotapes by eight trained graduate students in psychology. However, not all of these codes factored into the two parental skills considered to be theoretical important in the current study; for example, "Talk" which is neither positive nor negative was not addressed. Two sum-scores were generated: the sum-score of the parental Do Skills consisted of behavior descriptions, reflections and praises during CLP (ICC = .82), and the sum-score of parental Don't Skills consisted of negative talk, instructions and questions during CLP (ICC = .76). Child non-compliance was defined according to the manual [65] as a child response following a direct or indirect command given by the parent wherein the child does not perform, does not attempt to perform, or stops attempting to perform the requested behavior within the 5-second interval following the command. Non-compliance was calculated as the sum of their non-compliance to direct and indirect parental commands during PLP and CU (ICC = .58). The original English manual was used, because the coders spoke both English and Norwegian. The definitions, guidelines and examples from the English manual were mostly applicable to a Norwegian context. However, for some of the verbalizations, there was a need for an adaptation to the Norwegian language and culture, and in these cases Norwegian expressions and examples were created as supplementary material to the English DPICS-manual.

#### Preschool Age Psychiatric Assessment

Psychiatric disorders were assessed using the Preschool Age Psychiatric Assessment (PAPA [43]). The inter-rater reliability of the Norwegian PAPA used in this study has previously been found to be favorable, e.g., ODD κ = .89 and CD κ = .78 in the same age range and the same geographical location (Mid-Norway) as that of the present study [2].

## Results

Most of the children were diagnosed with a behavioral disorder after being assessed with the PAPA (Table 1) and half of the children had a concomitant emotional disorder. Diagnostic information was available only for 73 of the 81 participants due to technical problems with the recording of the interviews. Among the children with ODD or CD, 74.6% had at least one additional disorder (range, 0 to 7 disorders). The mean number of disorders was 2.7 (*SD* = 2.2). As shown in Table 1, many families had previously been in contact with child protective services.

Because the attrition analyses had revealed that the data were selective according to treatment and outcome (father’s ECBI), the data were missing not at random (MNAR). To accommodate intention-to-treat analyses, analyses addressing the effect of treatment were performed using Bayesian estimation, which is preferable to full information maximum estimation for small samples [66], under the assumption of missing completely at random using Mplus 7.31[67]. To test within-group differences between the initial testing and the two follow-ups, a model whereby the means at the initial testing and the 6-month assessment and the initial testing and 18-month follow-up, respectively, were set to be equal were compared to models where they were freely estimated, using the "model test" command in Mplus. The difference between models is tested with the Wald statistic. However, because Bayesian estimation is not conducive to this model test, a robust maximum likelihood estimator was used instead, which handled the data according to a full information maximum likelihood procedure. Gender, age, mothers’ and fathers’ scores on the CBCL, externalizing problems, and ECBI were used as auxiliary variables to increase the precision of the estimates if they were not already included as dependent or independent variables. At the initial assessment, the TAU and PCIT groups exhibited nearly identical ECBI scores (Table 2). The children in both the TAU and the PCIT groups evidenced improved ECBI and CBCL scores as rated by both mothers and fathers at both the 6-month assessment and the 18-month follow-up.

**Table 2:** Mean scores of behavior problems and parent-child interactions at the initial assessment, 6-month assessment and 18-month follow-up; ECBI, CBCL, Parental Do and Don’t Skills and Child Non-Compliance.

		Initial		6-month		Change from T1 to T2.		18-month.		Change from T1 to T3	
		assessment (T1)		assessment (T2)				follow-up (T3)			
		*M*	*SD*	*M*	*SD*	*Wald (p-value)*	*Hedges'g*	*M*	*SD*	*Wald (p-value)*	*Hedges' g*
ECBI Intensity—Mother	PCIT	147.87	20.58	122.65	11.51	21.01 (< .001)	1.49	120.50	30.82	13.39 (< .0001)	1.04
	TAU	155.13	24.25	132.38	32.78	8.39 (.004)	.79	136.21	29.80	8.44 (.004)	.70
ECBI Intensity—Father	PCIT	140.18	21.48	115.08	18.83	27.05 (< .001)	1.22	110.75	21.95	21.54 (< .0001)	1.34
	TAU	140.83	21.79	126.48	24.87	6.64 (0.1)	.61	121.18	30.48	15.24 (.0001)	.74
CBCL Externalizing -	PCIT	26.42	9.23	16.04	9.78	26.70 (< .001)	1.08	17.00	11.08	31.65 (< .0001)	.91
Mother	TAU	24.78	10.16	19.17	11.89	5.74 (0.17)	51	20.04	14.00	4.51 (.03)	.39
CBCL Externalizing -	PCIT	23.93	9.79	14.61	5.81	24.17 (< .001)	1.12	15.03	9.72	18.26 (< .0001)	.90
Father	TAU	20.70	9.43	16.59	9.44	4.61 (.03)	.43	15.65	11.45	8.94 (.003)	.48
Parental Do Skills	PCIT	4.43	3.35	16.34	7.43	55.19 (< .001)	-2.10	14.76	7.53	42.30 (< .0001)	-1.81
	TAU	6.02	3.51	5.44	3.09	.28 (.60)	.17	4.33	2.48	4.34 (.04)	.54
Parental Don’t Skills	PCIT	20.26	7.57	13.33	6.56	15.54 (< .001)	.96	10.09	3.37	30.04 (< .0001)	1.66
	TAU	21.27	6.68	22.42	5.43	.35 (.55)	-.18	16.22	6.89	6.76 (.009)	.74
Child Non-Compliance	PCIT	3.59	2.51	2.71	2.64	1.42	.34	2.12	3.33	3.16 (.07)	.50
	TAU	5.00	5.70	2.47	1.70	4.74	.55	3.78	5.72	.62 (.43)	.21

Note: ECBI = Eyberg Child Behavior Inventory, CBCL = Child Behavior Check list, PCIT = Parent-Child Interaction Therapy, TAU = treatment as usual, CI = confidence interval, T1 = initial assessment, T2 = 6-month assessment, T3 = 18-month follow-up.

To test whether the PCIT children improved more compared with the TAU group over the whole treatment and follow-up period (18 months), a linear latent growth curve analysis was conducted with growth parameterized as yearly change. One analysis was performed per outcome. At the 6-month assessment, fathers receiving PCIT rated their children’s behavior as less problematic compared with fathers receiving TAU per the ECBI (Table 3). Although the effects of treatment on CBCL ratings and mothers’ ECBI ratings did not reach significance, they were in the expected direction (i.e., favoring PCIT) and bordered on significance in the case of the mothers’ CBCL scores (*p* = .06). At the 18-month follow-up, the ECBI scores of the PCIT group did exhibit greater improvement compared with the TAU group, according to both the mothers and fathers. However, whereas the mother-rated CBCL scores also reflected greater improvement in the PCIT group, the improvement in father-rated CBCL scores did not reach significance.

**Table 3:** Effects of participating in PCIT versus TAU on child outcomes as an overall change from 0 to 18 months (growth curve analyses) and the prediction of outcomes at the 6- and 18-month assessments (linear regression).

	B [95% CI]	β	*p*-value	Cohen’s *d*
Overall change from 0 to 18 months				
ECBI Intensity—Mother	-10.12 [-22.13; 1.40]	-.31	.043	.64
ECBI Intensity—Father	-8.55 [-19.83; 2.26]	-.37	.062	.79
CBCL Externalizing—Mother	-3.08 [-6.48; 0.64]	-.29	.044	.61
CBCL Externalizing—Father	-1.63 [-5.42; 2.00]	-.22	.19	.44
Parental Do Skills	7.49 [5.02; 9.98]	.79	< .001	2.58
Parental Don’t Skills	-4.42 [-7.15; -1.67]	-.59	.001	1.46
Child Non-Compliance	-.79 [-2.62; 1.05]	-.25	.20	.52
Change from 0 to 6 months				
ECBI Intensity—Mother	10.87 [-3.77; 25.01]	.18	.11	.37
ECBI Intensity—Father	12.06 [.08; 26.75]	.27	.03	.56
CBCL Externalizing—Mother	4.29 [-.58; 10.06]	.19	.06	.39
CBCL Externalizing—Father	3.00 [-.98; 7.41]	.18	.095	.37
Parental Do Skills	-11.24 [-7.23; -15.58]	-.67	< .001	1.81
Parental Don’t Skills	9.61 [4.93; 14.04]	.58	< .001	1.42
Child Non-Compliance	.42 [-2.46; .98]	.09	.27	.18
Change from 6 to 18 months				
ECBI Intensity—Mother	17.00 [.15; 41.14]	.25	.02	.52
ECBI Intensity—Father	11.97 [.89; 25.89]	.19	.02	.39
CBCL Externalizing—Mother	5.46 [.98; 10.30]	.21	.01	.43
CBCL Externalizing—Father	3.38 [-2.12; 8.41]	.16	.11	.32
Parental Do Skills	-10.65 [-6.78; -13.99]	-.69	< .001	1.91
Parental Don’t Skills	5.64 [2.34; 9.68]	.39	< .001	.85
Child Non-Compliance	1.16 [-1.30; 3.99]	.11	.21	.22

Notes: PCIT = Parent-Child Interaction Therapy, TAU = treatment as usual; ECBI = Eyberg Child Behavior Inventory, CBCL = Child Behavior Checklist, CI = confidence interval. Intercepts are adjusted for in the growth curve analyses and initial values in the regression analyses.

Regarding observed parenting practices, parents in both groups increased their Do Skills and decreased their Don’t Skills after treatment. However, the increase in Do Skills and the decrease in Don’t Skills were substantially greater in the PCIT group compared with those that were in the TAU group. Additionally, there was a reduction in non-compliance among the children receiving PCIT bordering on significance (*p* = .07), but this change was not significantly different from that of the TAU children.

Hedges’*g* [68] was developed to eliminate the small positive bias affecting the calculation of the much-used Cohen’s *d* [69] when sample sizes are unequal between groups. At 6 months, a difference in-group sizes between the PCIT group and the TAU group appeared and we therefore calculated effect sizes with Hedges’ *g* when comparing means at 6-months and 18-month follow-up.

## Discussion

To examine the effectiveness of PCIT in routine service, we conducted an RCT with children receiving usual care as a comparison and followed up with the families 6 and 18 months after the start of therapy.

We hypothesized that the children receiving PCIT would demonstrate a greater reduction in behavior problems compared to children receiving TAU at follow-up, whereas parents who received PCIT would show greater improvement in parenting skills compared with parents who received TAU. Both of these hypotheses were supported. Overall, the PCIT children improved more compared with the TAU children when the whole treatment period was considered. However, the difference was first evident at the 18-month follow-up, when all families had completed therapy. The parents receiving PCIT improved their parenting skills more compared with the parents receiving TAU, and the children receiving PCIT evidenced fewer behavior problems compared with the children receiving TAU. However, even if the parents in the PCIT group improved their parenting skills to a greater extent compared with the parents in the TAU-group, the reduction in non-compliance among the children receiving PCIT was not significantly different from that of the TAU children. The results were comparable to those obtained in a study of Mexican-Americans using student therapists [38]. Accordingly, the present study extended the findings of the latter study of McCabe and colleagues, by showing that beneficial results of PCIT can also be obtained in regular clinical practice in a rather different cultural and health care context.

The lack of TAU and studies on usual care lead to PCIT being designated as probably efficacious [11]. However, the results from the two recent studies [17, 34], together with the present study, imply that the status of PCIT might be changed to efficacious. The results are also comparable or better in regards to reducing behavior problems in the three other studies using TAU: viz., *d* = .16 at post-treatment in a Norwegian study of PMT-O [13], although this improvement was no longer evident at the one-year follow-up [70]; *d* = .31 in an Icelandic PMT-O study using a composite of externalizing behaviors, internalizing behaviors, and social skills as outcomes ([13–18]; and, in contrast, no significant effect in an Irish parent training program [71].

As expected, the parenting skills of the PCIT group exhibited greater improvement compared with those of the TAU group, an improvement that was considerably greater compared with the average effect of parent training programs on parent skills acquisition (i.e., *d* = .30) [72]. The improvement observed among PCIT parents was nearly identical to that obtained in the McCabe et al. study [34] where PCIT was conducted by student therapists. In the present study, PCIT was not the major approach in the therapist’s regular clinical work; instead, the therapists were clinical practitioners who were trained in PCIT and had used the intervention as a part of their routine service prior to the trial.

The treatment effect should be considered in the context of TAU parents also improving in parenting skills, in contrast to the findings of McCabe and colleagues' PCIT study and the one PMT-O study to have reported improvement [13]. The strong effect of parental skill acquisition supports the fidelity of the PCIT treatment provided in the present study. Conceivably, the solid improvement observed in PCIT may be attributed to the facts that such parenting skills are directly targeted in PCIT and that therapy continues until these skills are mastered at pre-specified levels. It should be noted that the greater improvement in parenting skills and the reduction in behavioral problems evident in the PCIT group were obtained without the children being significantly less noncompliant, which is similar to results obtained in other studies [34, 70]. Because this study was conducted in Norway, the results might be influenced by the more permissive and democratic parenting styles suggested to be typical of the Nordic countries, [24]. Norwegian parents may be inclined to interpret non-compliance as an expression of a child's free will and independence and as typical developmental behavior rather than as a transgression, at least at moderate intensity and frequency. Hence, non-compliance may to a lesser degree instigate coercive cycles between parents and children in Norwegian families as opposed to families from other cultures, e.g. the U.S.A. Obviously this possibility needs to be examined through in-depth analyses of parent-child interactions. Moreover, there is a need for further research concerning whether the use of alternative back-up procedures in PDI will affect non-compliance. To our knowledge, no other PCIT studies have used the alternative back-up procedures used in this study. Hence, we cannot rule out that this might have affected the results with respect to non-compliance and potentially reduced the effect size. Conceivably, PCIT may have superior effect on child aggressiveness, emotion regulation and irritability compared with non-compliance.

Unlike many other parent programs designed to treat behavioral problems [73, 74], the present study recruited fathers as well as mothers, and fathers completed PCIT equally often as mothers. Although fathers, to a lesser degree compared with mothers, reported that their children’s behavior problems had diminished, their extended attendance, as suggested by others [75], might have contributed to the present positive long-term effect of PCIT.

### Limitations

The present results should be interpreted in the context of certain limitations. First, although the children were assessed for behavioral disorders prior to initiating treatment, we did not implement an equivalent diagnostic assessment after treatment. Thus, we cannot be sure that the present findings extend to ODD and CD. However, the ability of the CBCL to identify children with diagnosable behavioral disorders is fairly well established in the clinical setting [34, 76, 77], thus supporting the notion that the present findings may tentatively be extrapolated to ODD and CD. Therefore, it is acknowledged that such a proposition must be tested in future studies.

Second, it is well documented that pharmacotherapy reduces symptoms of ADHD [78]. Ten children in the present study received medication for ADHD, but because this was an RCT, the participants who used medication were randomly assigned to either PCIT or TAU, and supplementary analysis showed that there was no difference pursuant to assignment. The positive effect of medication should therefore have affected both groups in the same way.

Third, no formal measure of treatment fidelity was obtained. However, feedback to the therapists was offered on the basis of the PCIT protocol. Moreover, the improvement in parenting skills exhibited in the PCIT group was no less compared with studies where such fidelity checks were employed [34], thereby suggesting that the therapy was delivered according to the manual.

Fourth, there is a delicate balance between adherence to the intervention protocol and adherence to regular practice in clinics when conducting effectiveness studies in routine care. In this study, two therapists were present in 45% of the sessions for integrity purposes. However, this is above the standard in the participating clinics, where a co-therapist usually is present at the beginning of CDI and PDI but rarely beyond this standard, except for especially severe cases. Hence, the results might not fully generalize to regular practice in usual care.

Fifth, the dropout rates from PCIT-community studies suggest wide variations in rates of attrition, from 20% [30] to 67% [27]. In comparison, this study had an attrition rate from pre-test to the 6 and 18-month follow-ups of 28% and 20%, respectively. This is slightly greater compared with the Chaffin et al. study but clearly below the Lyon et al. study and the other European PCIT study, which had a 54% dropout rate [17]. In the present study, the participants with the most serious problems (ODD) had the lowest attrition rate, which differs from one other study, where higher cumulative risk predicted dropout [79] and where the families that needed help most were those that did not stay in therapy. However, differential attrition is not uncommon in studies using wait-list controls [32]; thus, one possible explanation for our relatively low dropout rate is the use of an active treatment for the control group. Moreover, weekly sessions place a great demand on families, and it is therefore conceivable that the families with the most serious problems were also the ones most motivated. Hence, the relatively low attrition rate might reflect that the families with the most serious problems—hopefully those that needed it most—were those who completed the intervention. Furthermore, the attrition rate might underscore the importance of conducting an active treatment control in effectiveness studies. Nonetheless, we cannot rule out that the results could have been different if more families had completed.

Finally, only a modest inter-rater reliability was obtained with regards to child non-compliance. Therefore, the lack of findings regarding change in non-compliance should thus be interpreted cautiously.

### Further research and implications for clinical practice

Parent training is one of the most effective treatments for young children with behavior problems, and there is sustainable evidence for psychosocial treatments as the treatment of choice [37]. However, few studies have evaluated the transportability of evidence-based programs to new settings and with different participant groups. The main purpose of the present study was therefore to provide intervention to treatment-seeking young children in a clinical service setting with regular therapists. To our knowledge, this is the first study of the effectiveness of PCIT that includes the full set of real-world criteria described by Michelson and colleagues [6] and Weisz and colleagues' [26] criteria for clinical representativeness with regard to participant enrollment and treatment setting. The study was also the first study of PCIT in Norway, and the participating clinics were the first to implement the intervention. In conclusion, the findings suggest that behavioral problems in children who receive PCIT in regular clinical practice will be reduced at moderate long-term follow-up to a greater extent compared with behavioral problems in children who receive TAU. Furthermore, parents who receive PCIT improve their parenting skills to a greater extent compared with parents who receive TAU.

In general, PCIT is well accepted among Norwegian families and therapists, and the intervention was implemented in regular clinics in Norway without modifications to the core components of PCIT. The therapists especially seemed to appreciate the individual tailoring of the treatment in addition to the participation of parents and children together in the sessions, while the families often highlighted the in-vivo coaching as useful. At present, PCIT is accepted as an intervention for young children with behavior problems in several CAMHS clinics in Norway. An important area for future research would be to assess the effect of PCIT as offered in regular clinics for internalizing disorders and to at-risk families.

Furthermore, even though the present effect sizes were on par with previous efficacy findings, the moderate size of these effects support the notion that clinic-referred populations likely to need additional support [15]. Further research on non-responders is therefore needed to optimize interventions. Moreover, there is a need to identify what type of additional services will be needed to increase the sustainability of the intervention. Finally, bearing in mind that we examined the indicators of behavioral problems on a continuous scale, it is important for future inquiries to ascertain whether PCIT also reduces the likelihood of behavioral disorders (i.e. ODD and CD).

## Supporting Information

S1 Text Effect of Parent Child Interaction Therapy (PCIT) in the treatment of behaviour disorders in small children. Trial Study Protocol.
(DOC)

S2 Text Effekt av Parent-Child Interaction Therapy (PCIT) i behandling av små barn med atferdsvansker. Prosjektbeskrivelse.
(DOC)

S3 Text CONSORT Checklist. **C**hecklist of information to include when reporting a randomised trial.
(DOC)

## References

[1] Wichstrøm L, Belsky J, Jozefiak T, Sourander A, Berg-Nielsen TS. Predicting Service Use for Mental Health Problems Among Young Children. Pediatrics. 2014;133(6):1054–60. 10.1542/peds.2013-3184 . 24819574

[2] Wichstrøm L, Berg-Nielsen TS, Angold A, Egger HL, Solheim E, Sveen TH. Prevalence of psychiatric disorders in preschoolers. Journal of Child Psychology and Psychiatry. 2012;53(6):695–705. 10.1111/j.1469-7610.2011.02514.x . 22211517

[3] Loeber R, Farrington DP. Young children who commit crime: Epidemiology, developmental origins, risk factors, early interventions, and policy implications. Development and Psychopathology. 2000;12(4):737–62. 10.1017/s0954579400004107 . 11202042

[4] Lundahl BW, Risser HJ, Lovejoy MC. A meta-analysis of parent training: Moderators and follow-up effects. Clin Psychol Rev. 2006;26(1):86–104. 10.1016/j.cpr.2005.07.004 . 16280191

[5] Fossum S, Handegard BH, Martinussen M, Morch WT. Psychosocial interventions for disruptive and aggressive behaviour in children and adolescents A meta-analysis. Eur Child Adolesc Psychiatry. 2008;17(7):438–51. 10.1007/s00787-008-0686-8 . 18427863

[6] Michelson D, Davenport C, Dretzke J, Barlow J, Day C. Do Evidence-Based Interventions Work When Tested in the "Real World?" A Systematic Review and Meta-analysis of Parent Management Training for the Treatment of Child Disruptive Behavior. Clin Child Fam Psychol Rev. 2013;16(1):18–34. 10.1007/s10567-013-0128-0 . 23420407

[7] Menting ATA, de Castro BO, Matthys W. Effectiveness of the Incredible Years parent training to modify disruptive and prosocial child behavior: A meta-analytic review. Clin Psychol Rev. 2013;33(8):901–13. 10.1016/j.cpr.2013.07.006 . 23994367

[8] de Graaf I, Speetjens P, Smit F, de Wolff M, Tavecchio L. Effectiveness of the Triple P Positive Parenting Program on Parenting: A Meta-Analysis. Family Relations. 2008;57(5):553–66. 10.1111/j.1741-3729.2008.00522.x . 18475003

[9] Sanders MR, Kirby JN, Tellegen CL, Day JJ. The Triple P-Positive Parenting Program: A systematic review and meta-analysis of a multi-level system of parenting support. Clin Psychol Rev. 2014;34(4):337–57. 10.1016/j.cpr.2014.04.003 24842549

[10] Nowak C, Heinrichs N. A comprehensive meta-analysis of triple p-positive parenting program using hierarchical linear modeling: Effectiveness and moderating variables. Clin Child Fam Psychol Rev. 2008;11(3):114–44. 10.1007/s10567-008-0033-0 . 18509758

[11] Eyberg SM, Nelson MM, Boggs SR. Evidence-based psychosocial treatments for children and adolescents with disruptive behavior. J Clin Child Adolesc Psychol. 2008;37(1):215–37. 10.1080/15374410701820117 . 18444059

[12] Thomas R, Zimmer-Gembeck MJ. Behavioral outcomes of parent-child interaction therapy and triple p-positive parenting program: A review and meta-analysis. J Abnorm Child Psychol. 2007;35(3):475–95. 10.1007/s10802-007-9104-9 . 17333363

[13] Ogden T, Hagen KA. Treatment effectiveness of Parent Management Training in Norway: A randomized controlled trial of children with conduct problems. J Consult Clin Psychol. 2008;76(4):607–21. 10.1037/0022-006x.76.4.607 . 18665689

[14] Sigmarsdottir M, Degarmo DS, Forgatch MS, Gudmundsdottir EV. Treatment effectiveness of PMTO for children's behavior problems in Iceland: Assessing parenting practices in a randomized controlled trial. Scand J Psychol. 2013;54(6):468–76. 10.1111/sjop.12078 . 24580570 PMC5308791

[15] Drugli MB, Larsson B, Fossum S, Morch WT. Five- to six-year outcome and its prediction for children with ODD/CD treated with parent training. Journal of Child Psychology and Psychiatry. 2010;51(5):559–66. 10.1111/j.1469-7610.2009.02178.x . 20015193

[16] Gerards S, Dagnelie PC, Gubbels JS, van Buuren S, Hamers FJM, Jansen MWJ, et al The Effectiveness of Lifestyle Triple P in the Netherlands: A Randomized Controlled Trial. PLoS One. 2015;10(4). 10.1371/journal.pone.0122240 . PMC4388496 25849523

[17] Abrahamse ME, Junger M, Wouwe MAMM, Boer F, Lindauer RJ. Treating child disruptive behavior in high-risk families: A comparative effectiveness trial from a community-based implementation. Journal of Child and Family Studies. 2015;11 26:No Pagination Specified. 10.1007/s10826-015-0322-4 PMC4824803 27110086

[18] Leung C, Tsang S, Sin TCS, Choi S-y. The Efficacy of Parent-Child Interaction Therapy With Chinese Families: Randomized Controlled Trial. Research on Social Work Practice. 2015;25(1):117–28. 10.1177/1049731513519827 .

[19] Lenze SN, Pautsch J, Luby J. Parent-Child Interaction Therapy Emotion Development: A Novel Treatment for Depression in Preschool Children. Depress Anxiety. 2011;28(2):153–9. 10.1002/da.20770 . 21284068 PMC3302425

[20] Puliafico AC, Comer JS, Pincus DB. Adapting Parent-Child Interaction Therapy to Treat Anxiety Disorders in Young Children. Child Adolesc Psychiatr Clin N Am. 2012;21(3):607–+. 10.1016/j.chc.2012.05.005 . 22800997

[21] Carpenter AL, Puliafico AC, Kurtz SMS, Pincus DB, Comer JS. Extending Parent-Child Interaction Therapy for Early Childhood Internalizing Problems: New Advances for an Overlooked Population. Clin Child Fam Psychol Rev. 2014;17(4):340–56. 10.1007/s10567-014-0172-4 . 25212716 PMC4258530

[22] Bagner DM, Coxe S, Hungerford GM, Garcia D, Barroso NE, Hernandez J, et al Behavioral parent training in infancy: A window of opportunity for high-risk families. J Abnorm Child Psychol. 2015;10 8:No Pagination Specified. 10.1007/s10802-015-0089-5 PMC4826322 26446726

[23] Kohlhoff J, Morgan S. Parent-Child Interaction Therapy for Toddlers: A Pilot Study. Child Fam Behav Ther. 2014;36(2):121–39. 10.1080/07317107.2014.910733 .

[24] Foros PB, Vetlesen AJ. Angsten for oppdragelse: Et samfunnsetisk perspektiv på dannelse Oslo, Norway: Universitetsforlaget; 2012.

[25] Weisz JR, Kuppens S, Eckshtain D, Ugueto AM, Hawley KM, Jensen-Doss A. Performance of Evidence-Based Youth Psychotherapies Compared With Usual Clinical Care A Multilevel Meta-analysis. Jama Psychiatry. 2013;70(7):750–61. 10.1001/jamapsychiatry.2013.1176 . 23754332 PMC3848075

[26] Weisz JR, Doss AJ, Hawley KM. Youth psychotherapy outcome research: A review and critique of the evidence base. Annu Rev Psychol. Annual Review of Psychology. 562005 p. 337–63. 10.1146/annurev.psych.55.090902.141449 15709939

[27] Lyon AR, Budd KS. A Community Mental Health Implementation of Parent-Child Interaction Therapy (PCIT). Journal of Child and Family Studies. 2010;19(5):654–68. 10.1007/s10826-010-9353-z . 20877583 PMC2945385

[28] Budd KS, Hella B, Bae H, Meyerson DA, Watkin SC. Delivering Parent-Child Interaction Therapy in an Urban Community Clinic. Cogn Behav Pract. 2011;18(4):502–14. .

[29] Pearl E, Thieken L, Olafson E, Boat B, Connelly L, Barnes J, et al Effectiveness of Community Dissemination of Parent-Child Interaction Therapy. Psychological Trauma-Theory Research Practice and Policy. 2012;4(2):204–13. 10.1037/a0022948 .

[30] Chaffin M, Funderburk B, Bard D, Valle LA, Gurwitch R. A Combined Motivation and Parent-Child Interaction Therapy Package Reduces Child Welfare Recidivism in a Randomized Dismantling Field Trial. J Consult Clin Psychol. 2011;79(1):84–95. 10.1037/a0021227 . 21171738

[31] Bagner DM, Eyberg SM. Parent-child interaction therapy for disruptive Behavior in children with mental retardation: A Randomized controlled trial. J Clin Child Adolesc Psychol. 2007;36(3):418–29. . 17658985 10.1080/15374410701448448

[32] Werba BE, Eyberg SM, Boggs SR, Algina J. Predicting outcome in parent-child interaction therapy—Success and attrition. Behav Modif. 2006;30(5):618–46. 10.1177/0145445504272977 . 16894233

[33] Schuhmann EM, Foote RC, Eyberg SM, Boggs SR, Algina J. Efficacy of parent-child interaction therapy: Interim report of a randomized trial with short-term maintenance. J Clin Child Psychol. 1998;27(1):34–45. 10.1207/s15374424jccp2701_4 . 9561935

[34] McCabe K, Yeh M. Parent-Child Interaction Therapy for Mexican Americans: A Randomized Clinical Trial. J Clin Child Adolesc Psychol. 2009;38(5):753–9. 10.1080/15374410903103544 . 20183659

[35] Eisenstadt TH, Eyberg S, McNeil CB, Newcomb K, Funderburk B. Parent-Child Interaction Therapy with Behavior Problem Children—Relative Effectiveness of 2 Stages and Overall Treatment Outcome. J Clin Child Psychol. 1993;22(1):42–51. 10.1207/s15374424jccp2201_4 .

[36] Nixon RDV, Sweeney L, Erickson DB, Touyz SW. Parent-child interaction therapy: A comparison of standard and abbreviated treatments for oppositional defiant preschoolers. Journal of Consulting and Clinical Psychology. 2003;71(2):251–60. 10.1037/0022-006x.71.2.251 . 12699020

[37] Comer JS, Chow C, Chan PT, Cooper-Vince C, Wilson LAS. Psychosocial Treatment Efficacy for Disruptive Behavior Problems in Very Young Children: A Meta-Analytic Examination. Journal of the American Academy of Child and Adolescent Psychiatry. 2013;52(1):26–36. 10.1016/j.jaac.2012.10.001 . 23265631 PMC4247988

[38] McCabe K, Yeh M, Lau A, Argote CB. Parent-Child Interaction Therapy for Mexican Americans: Results of a Pilot Randomized Clinical Trial at Follow-up. Behavior Therapy. 2012;43(3):606–18. 10.1016/j.beth.2011.11.001 22697448 PMC7194395

[39] Eyberg SM, Funderburk BW, Hembree-Kigin TL, McNeil CB, Querido JG, Hood KK. Parent-Child Interaction Therapy with behavior problem children: One and two year maintenance of treatment effects in the family. Child & Family Behavior Therapy. 2001;23(4):1–20. 10.1300/J019v23n04_01 .

[40] Hood KK, Eyberg SM. Outcomes of parent-child interaction therapy: Mothers' reports of maintenance three to six years after treatment. J Clin Child Adolesc Psychol. 2003;32(3):419–29. 10.1207/s15374424jccp3203_10 . 12881030

[41] Eyberg SM, Pincus DB. Eyberg Child Behavior Inventory and Sutter-Eyberg Behavior Inventory-Revised: Professional manual Odessa, Fl.: Psychological Assessment Resources; 1999.

[42] Reedtz C, Bertelsen B, Lurie J, Handegard BH, Clifford G, Morch WT. Eyberg Child Behavior Inventory (ECBI): Norwegian norms to identify conduct problems in children. Scand J Psychol. 2008;49(1):31–8. 10.1111/j.1467-9450.2007.00621.x 18190400

[43] Egger HL, Erkanli A, Keeler G, Potts E, Walter BK, Angold A. Test-retest reliability of the Preschool Age Psychiatric Assessment (PAPA). Journal of the American Academy of Child and Adolescent Psychiatry. 2006;45(5):538–49. 10.1097/01.chi.0000205705.71194.b8 . 16601400

[44] Webster-Stratton C, Hancock L. Training for parents of young children with conduct-problems: content, methods and therapeutic processes In: Briesmeister JM, Schaefer CE, editors. Handbook of parent training: Parents as co-therapists for childrens behaviour problems. New-York: Wiley; 1998.

[45] Patterson GR, Reid J, Dishion T. A social interactional approach: Antisocial boys 4: Castalia, Eugene, OR; 1992.

[46] DeGarmo DS, Patterson GR, Forgatch MS. How do outcomes in a specified parent training intervention maintain or wane over time? Prevention Science. 2004;5(2):73–89. 10.1023/B:PREV.0000023078.30191.e0 . 15134313

[47] Websterstratton C. Randomized trial of 2 parent-training programs for families with conduct-disordered children. J Consult Clin Psychol. 1984;52(4):666–78. 10.1037/0022-006x.52.4.666 . 6470293

[48] Beauchaine TP, Webster-Stratton C, Reid MJ. Mediators, moderators, and predictors of 1-year outcomes among children treated for early-onset conduct problems: A latent growth curve analysis. J Consult Clin Psychol. 2005;73(3):371–88. 10.1037/0022-006x.73.3.371 . 15982136

[49] Faul F, Erdfelder E, Buchner A, Lang A-G. Statistical power analyses using G*Power 3.1: Tests for correlation and regression analyses. Behav Res Methods. 2009;41(4):1149–60. 10.3758/brm.41.4.1149 . 19897823

[50] Organization WH. ICD-10 Psykiske lidelser og atferdsforstyrrelser In: helsetilsyn S, editor. Oslo: Gyldendal Norsk Forlag AS; 2000.

[51] Tripp G, Luk SL, Schaughency EA, Singh R. DSM-IV and ICD-10: A comparison of the correlates of ADHD and hyperkinetic disorder. J Am Acad Child Adolesc Psychiatry. 1999;38(2):156–64. 10.1097/00004583-199902000-00014 . 9951214

[52] Lahey BB, Pelham WE, Chronis A, Massetti G, Kipp H, Ehrhardt A, et al Predictive validity of ICD-10 hyperkinetic disorder relative to DSM-IV attention-deficit/hyperactivity disorder among younger children. Journal of Child Psychology and Psychiatry. 2006;47(5):472–9. 10.1111/j.1469-7610.2005.01590.x . 16671930

[53] Integrity checklists and session materials. Parent-child interaction therapy manual [Internet]. 1999.

[54] Eyberg SM, Funderburk B. Parent-child interaction therapy. Integrity checklists and session materials. Gainesville, FL.: www.PCIT.org; 1999.

[55] McNeil CB, Hembree-Kigin TL. Parent-Child Interaction Therapy. New York: Springer; 2010

[56] Hembree-Kigin TL, McNeil CB. Parent-child interaction therapy New York: Springer; 1995.

[57] Hedenbro M, Wirtberg I, editors. Samspillets kraft; Marte Meo—mulighet til utvikling Oslo: Kommuneforlaget; 2002.

[58] Webster-Stratton C, Reid MJ, Hammond M. Treating children with early-onset conduct problems: Intervention outcomes for parent, child, and teacher training. Journal of Clinical Child and Adolescent Psychology. 2004;33(1):105–24. 10.1207/s15374424jccp3301_11 . 15028546

[59] Larsson B, Fossum S, Clifford G, Drugli MB, Handegard BH, Morch WT. Treatment of oppositional defiant and conduct problems in young Norwegian children Results of a randomized controlled trial. Eur Child Adolesc Psychiatry. 2009;18(1):42–52. 10.1007/s00787-008-0702-z . 18563473

[60] Achenbach TM, Rescorla L. Manual for the ASEBA preschool forms and profiles Burlington, VT: University of Vermont; 2000.

[61] Achenbach TM, Rescorla LA. Manual for the ASEBA school age forms and profile Burlington: University of Vermont Research Center for Children, Youth and Families; 2001.

[62] Jozefiak T, Larsson B, Wichstrom L, Rimehaug T. Competence and emotional/behavioural problems in 7-16-year-old Norwegian school children as reported by parents. Nordic Journal of Psychiatry. 2012;66(5):311–9. 10.3109/08039488.2011.638934 . 22171934

[63] Achenbach TM, Becker A, Doepfner M, Heiervang E, Roessner V, Steinhausen H-C, et al Multicultural assessment of child and adolescent psychopathology with ASEBA and SDQ instruments: research findings, applications, and future directions. Journal of Child Psychology and Psychiatry. 2008;49(3):251–75. 10.1111/j.1469-7610.2007.01867.x . 18333930

[64] Novik TS. Child Behavior Checklist item scores in Norwegian children. Eur Child Adolesc Psychiatry. 2000;9(1):54–60. . 10795856 10.1007/s007870050116

[65] Eyberg SM, Nelson MM, Duke M, Boggs SR. Manual for the Dyadic Parent-Child Interaction Coding System. 3. ed. Gainesville 2005.

[66] van de Schoot R, Kaplan D, Denissen J, Asendorpf JB, Neyer FJ, van Aken MAG. A Gentle Introduction to Bayesian Analysis: Applications to Developmental Research. Child Dev. 2014;85(3):842–60. 10.1111/cdev.12169 . 24116396 PMC4158865

[67] Muthén LK, Muthén BO. Mplus User's Guide. 7th ed. Los Angeles, CA: Muthén & Muthén; 1998–2015.

[68] Ellis PD. Effect size equations 2009 [cited 2015 03.01].

[69] Hedges LV. Distribution theory for Glass estimatior of effect size and related estimators. Journal of Educational Statistics. 1981;6(2):106–28.

[70] Hagen KA, Ogden T, Bjornebekk G. Treatment Outcomes and Mediators of Parent Management Training: A One-Year Follow-Up of Children with Conduct Problems. J Clin Child Adolesc Psychol. 2011;40(2):165–78. 10.1080/15374416.2011.546050 . 21391015

[71] Griffin C, Guerin S, Sharry J, Drumm M. A multicentre controlled study of an early intervention parenting programme for young children with behavioural and developmental difficulties. Int J Clin Health Psychol. 2010;10(2):279–94. .

[72] Lindhiem O, Higa J, Trentacosta CJ, Herschell AD, Kolko DJ. Skill Acquisition and Utilization During Evidence-Based Psychosocial Treatments for Childhood Disruptive Behavior Problems: A Review and Meta-analysis. Clinical Child and Family Psychology Review. 2014;17(1):41–66. 10.1007/s10567-013-0136-0 . 23649324 PMC3859702

[73] Lundahl BW, Tollefson D, Risser H, Lovejoy MC. A meta-analysis of father involvement in parent training. Research on Social Work Practice. 2008;18(2):97–106. 10.1177/1049731507309828 .

[74] Coplin JW, Houts AC. Father involvement in parent training for oppositional child-behavior—progress or stagnation. Child Fam Behav Ther. 1991;13(2):29–51. .

[75] Bagner DM, Eyberg SM. Father involvement in parent training: When does it matter? J Clin Child Adolesc Psychol. 2003;32(4):599–605. 10.1207/s15374424jccp3204_13 . 14710469

[76] Krol N, De Bruyn EEJ, Coolen JC, van Aarle EJM. From CBCL to DSM: A comparison of two methods to screen for DSM-IV diagnoses using CBCL data. J Clin Child Adolesc Psychol. 2006;35(1):127–35. 10.1207/s15374424jccp3501_11 . 16390308

[77] Brasil HHA, Bordin IA. Convergent validity of K-SADS-PL by comparison with CBCL in a Portuguese speaking outpatient population. BMC Psychiatry. 2010;10 10.1186/1471-244x-10-83 . PMC2984471 20955616

[78] Connolly JJ, Glessner JT, Kao C, Elia J, Hakonarson H. Attention-Deficit Hyperactivity Disorder and PharmacotherapyPast, Present, and Future: A Review of the Changing Landscape of Drug Therapy. Therapeutic Innovation & Regulatory Science. 2015;49(5):632–42. 10.1177/2168479015599811 . 26366330 PMC4564067

[79] Bagner DM, Graziano PA. Barriers to Success in Parent Training for Young Children With Developmental Delay: The Role of Cumulative Risk. Behav Modif. 2013;37(3):356–77. 10.1177/0145445512465307 . 23188886 PMC4479170

