# Supplementary material for: Effectiveness of Parent-Child Interaction Therapy (PCIT) in the Treatment of Young Children’s Behavior Problems. A Randomized Controlled Study
Source: PLoS One. 2016 Sep 13;11(9):e0159845. doi: 10.1371/journal.pone.0159845 (PMC5021353; doi:10.1371/journal.pone.0159845)
Supplement: S1 Text — Trial Study Protocol. (DOC) [file pone.0159845.s001.doc]

**Effect of Parent Child Interaction Therapy (PCIT) in the treatment of behaviour disorders in small children**

**Trial Study Protocol**

Åse Bjørseth, ClinPsyD

Levanger Hospital, Nord-Trøndelag Hospital Trust, Norway

Department of Psychology, Norwegian University of Science and Technology

Anne Kristine Wormdal

Associated Professor/Clinical psychologist

Department of Psychology, Norwegian University of Science and Technology

St. Olav’s Hospital, Trondheim University Hospital

Lars Wichstrøm, Ph.D., Professor

Department of Psychology, Norwegian University of Science and Technology

**Background**

***Parent-Child Interactiona Therapy (PCIT)***

The intervention was developed by the psychologist Sheila Eyberg. It was originally developed as an intervention for small children with behavior problems, i.e. oppositional defiant disorder (ODD) and hyperactivity (ADHD). PCIT is strongly influenced by social learning theory, non-directive play therapy, attachment theory and family therapy. A central assumption behind PCIT is that young children whose parents demonstrate a high degree of warmth, responsiveness and sensitivity to their signals, are more likely to develop a more secure working model of their relationship with others and to develop more effective emotional regulation. PCIT therefore consists of two phases. Parents are first taught a set of skills that promote a nurturing and secure relationship with their child, and then a second set of skills designed to increase the child’s prosocial behavior and decrease negative behavior.

***Treatment of small children with disruptive behaviour.***

Disruptive behaviour is the most common reason for referral to child mental health services both internationally (Kazdin, 1995) and in Norway (Bjørngård, 2001). In addition to high prevalence, disruptive behaviour in young children has been demonstrated to have high stability and it is a predictor of serious problems that continue across stages of development. It can lead to a wide range of psychiatric disorders in adulthood as well as delinquent and criminal behaviour (Carr, 1999).

This has been the background for a concerted effort to find and develop treatments that are effective in not only producing immediate positive change in children’s behaviour, but that also show long-term maintenance of these gains. In Norway there has been a joint effort by several governmental ministries to give financially support to the implementation of three new methods: Webster Stratton’s “The incredible years” (Webster-Stratton & Hancock, 1998), Multisystemic Therapy (MST; Henggeller, Melton & Smith, 1992) and Parent Management Training-Oregon (PMT-O; Ogden, 2000).

This study concerns Parent-Child Interaction Therapy (PCIT), a method that has been empirically supported to be effective in treatment of young children with behaviour disorders in USA, PCIT has so far not been widely known or used in Norway, but in recent years a group of therapists in mental health clinics in Sør- and Nord-Trøndelag have started training and using PCIT on a more explorative basis.

Several studies have shown that PCIT is effective in reducing disruptive behaviour at home and in the family (Eisenstadt et al, 1993; Eyberg et al, 1995), and that treatment with PCIT leads to changed behaviour at school (McNeil et al, 1991). Parents also report changes in untreated siblings (Brestan et al. 1997). A recent follow-up study suggests that treatment effect is maintained 6 years after treatment (Hood & Eyberg, 2003).

Eyberg’s (1998) study shows that PCIT is most effective when used with children between 2 and 8 years of age. It is assumed that this is partly due to the use of play as a mean of changing the interaction between parent and child, and that playing is especially important for the child in these years. Factors that seem to reduce the efficiency are strong conflicts between the parents, substance abuse, severe learning disabilities and severe psychopathology with the parents (Eyberg & Boggs, 1998).

Theoretically PCIT has a common foundation with PMT and Webster Stratton’s methods in both social learning theory and attachment theory. The main differences between the methods are in the format of the therapy. PCIT is characterized by live coaching via ear-phones of individual parents during play with their child. The use of live coaching of a parent while the parent and child play together makes it different from any of the other methods. The use of play instead of talking makes it different from PMT, and can suggest that this is a method that is especially suited for small children. PCIT differs from Webster-Stratton’s method in that it is used with individual families. This can make it possible to adjust the treatment to the needs of a broader range of families, and it is suggested that it can better address behaviour disorder that when it is seen as co-morbidity with other disorders like ADHD and disorders in the autism spectrum.

PCIT is today an intervention in use in several Child and Adolescent Mental Health Clinics in Mid-Norway. There is established a task force, that have helped developing and supporting this study. Continuous evaluation of the clinical practice at these clinics has served as a pilot study for this study. There is also established a cooperation with researchers in the USA, and one of this group of researchers will serve as supervisor for the trial.

***Theory/Research***

Today it is widely accepted that a dysfunctional interaction between parent and child is central in development of behaviour disorders in children (Campbell, 1990; Patterson, 1982). This has led to development of interventions where these patterns of interactions are changed. Family interventions and parent training has been established as superior to other interventions as evidence based treatment of behaviour disorders (McClellan & Weary, 2003).

Behan and Carr (2000) identified 24 studies of effect of treatment and concluded that behaviourally oriented parent training combined with training of problem solving for the children were most effective for children diagnosed as having Oppositional Defiant Disorder (ODD). ODD is the most common form of behaviour disorder with smaller children while Conduct Disorder (CD) is most common with adolescents. ODD is a strong risk factor for development of CD.

Gallagher (2003) has examined all studies done on effect of PCIT. She concludes that the studies give strong support to PCIT as an effective intervention, and that it can be recommended as an evidence-based treatment for young children with behaviour disorders. However, she points out, there could be a limitation to these findings in that many of the studies are done in university clinics where students get extensive training and the studies are done as a part of their doctoral work. This could make it more difficult to know if the results would be the same in a more realistic clinical setting.

So far, the evidence for PCIT is categorized as “Probably efficatious treatment” (Hibbs, 2001). The reason that it is not categorized as a well-established psychosocial intervention is that only one study of effect has used alternative treatment as control-group, while the others have used waiting-list controls. This study is based on alternative treatment as control-group.

**Purpose of this study**

The primary purpose of this study is to evaluate effect and efficiency of PCIT in ordinary mental health clinics in Norway.

***Research-questions*:**

1. Will symptoms of behaviour disorder be reduced and the prevalence of this diagnosis be lower in a group of children between 2 to 8 years who have been treated with PCIT compared to children and families who receive standard treatment.
2. To what degree will this effect be maintained at 6 and 18 months follow-up?
3. To what degree will parent’s behaviour toward their children change as registered by the DPICS?
4. Will effect of treatment be modified or mediated by parent’s experienced locus of control or depressive symptoms?
5. Will there be effect of treatment when PCIT is used as a short-term intervention during three-week in-patient treatment for families?
6. Will parent’s quality of life improve as an effect of reduction in the child’s behavior problems?
7. Will the child’s pattern of attachment change as a result of treatment?
8. Will family functioning improve as an effect of reduction in the child’s behavior problems?

**Method**

***Participants*.**

Children between 2 and 8 years that are referred to one of the five participating clinics in Trøndelag and to the mental health clinic for children at Department of psychology at the university in Trondheim - NTNU.

***Criteria for inclusion:***

- Child’s age is between 2 and 8 at intake.
- Score of 120 or more on ECBI intensity scale or diagnosed as having a disruptive behavior disorder.
- Family has gone through screening procedure and has signed an agreement to participate.

***Criteria for exclusion***:

- Parental severe psychiatric disorder or drug-abuse.
- Suspected abuse or serious maltreatment.
- Child is severely mentally retarded.

**Procedure**

All children between 2 and 8 that are referred to the participating clinics with symptoms of disruptive behaviour or symptoms of hyperactivity or attention-deficit are screened with ECBI (Eyberg & Pincus, 1999). After deciding if the child/family can be included in the project, they will be allocated to either control or treatment group. Families will be evaluated at start of treatment, by termination of treatment and 18 months after start of the treatment. The length of the treatment with PCIT will vary depending on how long it will take for parents to meet the criteria that are set for mastery of the new skills. All families will join the project when they are ready for treatment, so the treatment group will not be given treatment earlier than the control group.

For some conditions, especially ADHD, and sometimes also for ADD, there will be an indication for medication. When medication is indicated it will be administered according to ordinary routines in the clinics. From ethical considerations, this will apply to both the treatment- and the control group.

The treatment manual (McNeil & Eyberg, 2004), has been translated into Norwegian by Wormdal (2005). The skills, the capacity and the treatment fidelity of the participating therapists are critical factors in this study. Therapists at the participating clinics have been given adequate training in the intervention, and the treatment will be provided according to the protocol.

**Design**

RCT-study. Treatment group (n=35) will receive PCIT as treatment. Control group (n=35) will receive what is used as standard treatment in that clinic. This treatment will vary somewhat between therapists and clinics, and could include counselling to parents, family therapy play therapy or individual cognitive therapy. Medication (usually stimulantia like Ritalin) and counselling to school or kindergarten will be offered to both groups if indicated. It will be examined whether these additional treatments are factors that are strong enough to influence possible differences between treatment and control group.

Clinics that offer Webster-Stratton or PMT-O as standard treatment will not be included in the study.

Random allocation to the groups will be done by using SPSS.

**Study plan**

The following child and adolescent mental health clinics are included in the study, and are planned to contribute with the described numbers of participants:

| **Clinic** | **PCIT-therapists** | **In training** | **PCIT-group** | **Control-group** |
| --- | --- | --- | --- | --- |
| Røros | 1 | 1 | 5 | 5 |
| Orkdal | 3 |  | 10 | 10 |
| Fosen | 1 | 1 | 5 | 5 |
| Levanger | 2 |  | 10 | 10 |
| Namsos | 1 | 1-2 | 5 | 5 |

The participating clinics have installed the necessary technical equipment. Continous training of PCIT-therapists will ensure that there are at least two PCIT-therapists in each of the participating clinics.

##### Organisation and critical factors

An important part of the study will be to organize for the PCIT-therapists to deliver therapy of high quality. The therapists at the participating clinics have been given adequate training and will provide the treatment according to the protocol. Nevertheless, the skills, the capacity and the treatment fidelity of the participating therapists are critical factors in this study. It will also be necessary to train new therapists during the trial. As the participating therapists will work in different clinics, they will meet regularly for supervision. Treatment integrity will be evaluated continuously and the inter-rater reliability of the coders of the parent-child interaction will be checked according to standards (Eyberg et al., 1994).

**Statistical strength.**

Effect-size for PCIT seems to be close to what has been found using Webster-Stratton’s method. The number of subjects in this study will therefore be based on what has been used in earlier studies. The ECBI is a continuous measure, which will increase the sensitivity to differences in effect-sizes between the experiment and control-group. With the present number of referrals to these clinics, and with an estimated percentage of parents who do not want to participate in the study, it is estimated that the treatment-phase will be at least two years. When the range is assumed to be equal in the experiment and the control group, the number of participants is set to be 35 in each group. Inclusion of more clinics in the study will reduce this period.

**Blindness**

ECBI and K-SADS/PAPA are done initially before allocation to a group. Video-recordings of interaction between parent and child will be made at the clinic for coding before and after treatment. Coders will be persons that have been trained by the Webster-Stratton clinic. This clinic is not included in the study and the coders will be “blind”. K-SADS-interviews will be done by staff from the participating clinics, but it will be done before allocation to a group.

**Measurements**

***Assessment of the child***

**Anamnestic assessment.** According to the practice at the participating clinics.

**ASEBA**. Child Behaviour Check List (CBCL) and Teacher Rating Form (TRF) will be used. These measurements are used as standard registration before and after treatment in the clinics (Achenbach, 1992).

**K-SADS-PL** (Kaufman, 1997) Kiddie-SADS-PL has been translated to Norwegian at R-BUP, NTNU. It is now widely used at clinics in this region, where a number of the staff has been trained to do this diagnostic interview. The interview is used in an epidemiologic study in the region (Sund, 2003).

**The Preschool Age Psychiatric Assessment (PAPA)**. Psychiatric disorders were assessed using the structured interview the Preschool Age Psychiatric Assessment for assessing and diagnosing psychiatric disorders for preschool children. The interview is translated to Norwegian, and training of the interviewers will be conducted September 2006 (Berg- Nilsen og Wichstrøm). PAPA will be used as pre-treatment assessment as soon as the training is finished.

**ECBI (Eyberg Child Behaviour Inventory).** Has been standardized in Norway as a part of the Webster-Stratton study. It is a recommended and widely used inventory of behaviour disorders in children.

**Working Model of the Child Interview** (Rosenblum, Zeanah, McDonough & Muzic, 2004). Parent interview that describes the child’s attachment pattern in relation to the interviewed parent.

***Assessment of Parent-Child Interaction***

**DPICS (Dyadic Parent-Child Interaction Coding System)** (Eyberg et al, 1994). This is a coding system for registration of parent-child interaction based on video-recordings or observation. A video-recording of parent-child interaction will be made before and after treatment. In addition each PCIT session will start with a 5 minute observation and coding by the therapist of the interaction. There is as mentioned earlier a number of trained coders that can blindly code the video-recordings.

***Assessment of Parent Functioning***

**Beck Depression Inventory (BDI)**. A possible connection between parental (maternal) depression and behaviour disorders has been noted earlier. This can both be caused by less sensitivity to the child or to reduced confidence as parent with a difficult child. Hood and Eyberg (2003) found, however, no significant changes in BDI measured before and after treatment.

**PLOC-SF (Parental Locus of Control-Short Form)**. This is an inventory for parents to measure their perception of being in control and being able to master the child’s behaviour. It has been demonstrated to be fairly sensitive to changes and effects of treatment (Campis, Lyman & Prentice-Dunn, 1986).

***Assessment of Family Functioning***

**Family Assessment Measure (FACES-IV).**

**Treatment procedure for PCIT**

The procedure has been described in a new manual (McNeil & Eyberg, 2004) and in a book by Hembree-Kigin and McNeil (1995). A Norwegian translation of the manual has been made by Wormdal (2005).

***Evaluation and preparation.***

Before treatment an assessment will be done that also includes interviews with the child’s parents about the problems that the child and the family has. The aim of this is to find goals for the treatment together with the parents. It is recommended to work with fairly wide goals, as the aim of the treatment is to change interaction patterns in the family, not specific behaviours.

***Step 1: Child Directed Interaction (CDI).***

The first part of the treatment starts with a didactic session where the parents come alone, and the therapist introduces the skills set that will be learned in this phase. Role-play is often used to demonstrate skills and the coaching situation to parents. Subsequently the child and parents come to the weekly sessions together. At the beginning of each session there is first a short talk with parents about homework in the previous week. When the child has two parents they are coached one at a time while playing. The coaching begins with a 5-minute observation of the interaction that is coded with DPICS, and the therapist gives immediate feedback from this observation to the parent.

The parent is instructed to engage in “special play time” with their child where they use elements from play therapy. This means avoid asking questions, to avoid criticism (saying “no”, “stop” etc.) and to avoid commands. Instead they are told that they will be coached to use the PRIDE-skills: **P**raise appropriate behaviour, **R**eflect what the child says, **I**mitate appropriate play, **D**escribe appropriate behaviour and be **E**nthusiastic. A hand-out describing these “Do” and “Don’ts” and the rationale for them is given to the parents.

Coaching is given as short precise comments from the therapist to the parent so as to not disturb the flow of the interaction with the child. In these comments the therapist will focus on the parent’s mastery, to be encouraging, to give gentle suggestions and to point to how what happens in the session will have effect on the child’s behaviour. Often the other parent stays with the therapist in the observation room, and the therapist can explain and have some interaction with this parent at the same time. After about 20 minutes coaching the parents change place. At the end of the session there is another short conversation with both the parents and the parents are encouraged to practice new skills in daily 5-minutes special play-time at home.

Before the next step of the therapy the parents must reach mastery of the CDI-skills. Criteria for mastery are set high to ensure that these skills become more or less automatic for the parents. With some families, the child’s behaviour will have changed so much during the CDI-phase that it is not necessary to go to the next step. Normally, however, it is seen that while CDI increases positive behaviour, PDI is the part that reduces negative behaviour in both children and parents.

***Step 2: Parent Directed Interaction (PDI).***

This next phase also starts with one or two didactic sessions for the parents. The parents will be trained in how instructions to the child should be given, and the importance of being consistent and predictable is underlined. It is also stressed that it is important to stay calm and in control of own reactions during limit setting. Parents are often trained and instructed in using anger-control techniques in this process. The child is also informed that the next step is exercises in minding the parents, and the child is shown how this will be done in role-play and often by using dolls. Training consists of minding exercises, real-life exercises, training by having another sibling in the same room, establishing house-rules etc. At the same time the skills that are learned in the CDI-phase are used in the interaction with the child to further strengthen the relationship and to reduce anger and conflict.

**Time-schedule**

The study is planned to start 01.03.06 and continue until 31.02.08. Recruiting of new families to the study will take place during the first two years. Data obtained until 31.12.07 will be included in the study. To fasten reporting, trial-analyses of the material will be made and writing of reports will start before the final data are collected.

**Clinical and scientific value of the study**

As shown earlier, the costs of having behaviour disorders are very high for both families, the individuals and for society. Interventions that can effectively reduce these problems are needed. If it is possible to establish trough this study that PCIT is efficient in reducing behaviour when used with small children in Norway, a new method will be available for the mental health care service. PCIT has a format and a structure that makes it easy to implement in smaller mental health care clinics. In a country like Norway, with a small and scattered population and many small clinics, this could mean that an efficient intervention would become available to more people. It would be a valuable supplement to other methods like PMT-O and Webster-Stratton. In addition this would give PCIT status as an internationally documented efficient treatment.

**Ethical considerations.**

Parents will get written and oral information along with a written invitation to participate in the project, and they will then be asked to give a written consent to participate. From the preliminary use of PCIT we know that parents can feel that the initial part of the therapy, and exposing their interaction with the child directly to the therapist, is demanding. This changes greatly when they develop more confidence in the therapist. The children seem to find the first part (CDI) very pleasant because it gives them a lot of positive attention and support from their parents. For them the second part of the therapy, with the minding exercises and limit setting, seems to be more demanding. But most likely the situations that arise in the therapy-room are more controlled than the limit setting and conflicts they experience every day at home.

Other documented treatments like medication for children with diagnosed ADHD will be given to children in both groups if indicated. Also counselling to school and kindergarten will be provided to both groups if indicated.

**Publication - dissemination.**

Results from the study will be presented in articles that will be submitted for publication in international journals in the field of clinical child psychology. The number of articles will meet the demands for a Ph.d, that is four articles submitted for publication in international journals with peer-review. The data collected can also be used for further analysis and more publications later. The results will also be presented at national and international conferences in the field.

**Financing**

It is predicted that the participating clinics will cover costs connected to training and supervision of the therapists and payroll expences for the therapists, in addition to necessary tecnical equipment to provide the therapy. We will apply to get payroll expences for the coders financed from other sources.

**References**

Achenbach, T. M. (1992). Manual for the Child Behaviour Check List/2-3 and 1992 profile.

Burlington: University of Vermont, Department of Psychiatry.

Behan, J. & Carr, A. (2000) Oppositional defiant disorder. I: A.Carr (Ed.) *What works with Children and adolescents.* London: Routledge.

Bjørngaard, J.H. (2001) *Samdata Psykisk helsevern*. Tabeller. SINTEF Unimed, Rapport 2001.

Bjørseth, Å. & Wormdal, A.K. (2005) Parent Child Interaction Therapy - Med terapeuten på øret. *Tidsskrift for Norsk Psykologforening*, 8, 2005*.*

Brestan, E., Eyberg, S., Boggs, S., & Algina, J. (1997) Parent Child Interaction therapy. Parent perceptions of untreated siblings. *Child and Family Behavior Therapy*, 19, 13-28.

Campis, L.K., Lyman, R.D. & Prentice-Dunn, S. (1986) The Parental Locus of Control Scale: Development and Validation. Journal of Clinical Child Psychology, Vol.15, 1986.

Carr, A. (1999) *The handbook of child and adolescent clinical psychology. A contextual approach.* London: Routledge.

Eisenstadt, T.H., Eyberg, S., McNeil, C.B., Newcomb, K., & Funderburk, B. (1993) Parent-child interaction therapy with behaviour problem children: Relative effectiveness of two stages and overall treatment outcome. *Journal of Clinical Child Psychology*, 22. 42-51.

Eyberg, S., Boggs, S., & Algina, J. (1995) Parent-child interaction therapy. A psychosocial model for treatment of young children with conduct problem behavior and their families. *Psychopharmacology Bulletin*, 31, 83-91.

Eyberg, S. & Pincus, D. (1999) Eyberg Child Behavior Inventory and Sutter-Eyberg Student Behavior Inventory: Professional manual. Odessa,FL: Psycholocical Assessment Resources.

Eyberg, S.M, Nelson, M. D., Duke, M. & Boggs, S. R.(2005). Manual for the Dyadic parent-child interaction coding system. University of Florida: http://www.pcit.org

Eyberg, S.M. & Boggs, S.R. (1998). Parent-child Interaction therapy: A psychosocial intervention for the treatment of young conduct-disordered children. In J.M. Briesmeister & C.E. Schaefer, (Eds.) *Handbook of parent training: Parents as co-therapists for children`s behavior problems*. New York: Wiley.

Eyberg, S.M., (1998) Parent-Child Interaction Therapy. Intergration of traditional and behavioral concerns. *Child and Family Behavior Therapy,10*, 33-46.

Gallagher, N. (2003) Effects of Parent-Child Interaction Therapy on Young Children with Disruptive Behaviour Disorders. *Bridges*, Vol.1, No. 4.

Hembree-Kigin, T. & McNeil, C.B. (1995). *Parent–Child Interaction Therapy.* New York: Plenum.

Henggeler, S. W., Melton, G. B., & Smith, L. A. (1992). Family preservation using multisystemic therapy: an effective alternative to incarcerating serious juvenile offenders. Jo*urnal of the American Academy of Child and Adolescent Psychiatry, 41*, 868 – 874.

Hood, K. K. & Eyberg, S. M.(2003). Outcomes of Parent-Child Interaction Therapy: Mothers’ Reports of Maintenance Three to Six Years after Treatment. *Journal of Clinical Child and Adolescent Psychology*, 2003, Vol. 32, No. 3.

Hibbs, E.D. (2001) Evaluating empirically based psychotherapy research for children and adolescents. *European Child & Adolescent Psychiatry*, 10, 2001.

Kaufman, J. et al. (1997) *Kiddie-SADS-PL. Schedule for Affective Disorders and Schizophrenia for School Aged Children – Present Life Version*.

Kazdin, A. E. (1995) *Developmental clinical psychology and psychiatry: Vol. 9. Conduct disorders in childhood and adolescence*. Thousand Oaks, CA: Sage.

McClellan, J. M. & Werry, J.S. (2003) Evidence-Based Treatments in Child and Adolescent Psychiatry: An inventory. *Journal of the American Academy of Child & adolescent Psychiatry*, Vol. 42(12)

McNeil, C. B. et al. (1991) Parent-Child Interaction Therapy with behavior problem children: Generalization of treatments effects to the school setting. *Journal of Clinical Child Psychology* 20: 140-151.

Ogden, T. Alvorlige atferdsproblemer hos barn – på jakt etter nye løsninger. *Barnevernshåndboka*, 2000.

Patterson, G.R. (1982) *Coercive family process*. Eugene, OR: Castalia.

Rosenblum, K.L., Zeanah, C., McDonough, S. & Muzik, M. (2004). Video-taped coding of

working model of the child interviews: a viable and useful alternative to verbatim transcripts?

*Infant Behavior & Development*, *27*, 544–549.

Webster-Stratton, C. & Hancock, L. (1998). Training for parents of young children with conduct problems: content, methods, and therapeutic processes. In J. M. Briesmeister,

& C. E. Schaefer, (Eds.) *Handbook of parent training: Parents as co-therapists for childrens behaviour problems*. New York: Wiley.

Wormdal, A. K. (2005) Manual for Parent-Child Interaction Therapy. Unpublished manuscript.
