# Supplementary material for: Effectiveness of Parent-Child Interaction Therapy (PCIT) in the Treatment of Young Children’s Behavior Problems. A Randomized Controlled Study
Source: PLoS One. 2016 Sep 13;11(9):e0159845. doi: 10.1371/journal.pone.0159845 (PMC5021353; doi:10.1371/journal.pone.0159845)
Supplement: S2 Text — Prosjektbeskrivelse. (DOC) [file pone.0159845.s002.doc]

**Effekt av Parent Child Interaction Therapy (PCIT) i behandling av små barn med atferdsvansker**

# Prosjektbeskrivelse

Åse Bjørseth

psykologspesialist/stipendiat

Familieenheten BUP

Sykehuset Levanger

Anne Kristine Wormdal

psykologspesialist/universitetslektor

Psykologisk institutt, NTNU, Trondheim

Barne- og ungdomspsykiatrisk klinikk, St. Olavs hospital, Trondheim

Lars Wichstrøm

professor

Psykologisk institutt, NTNU, Trondheim

**Bakgrunn**

***Parent-Child Interaction Therapy***

Behandlingsmetoden er utviklet av den amerikanske psykologen Sheila Eyberg. Den ble opprinnelig utviklet for å behandle små barn med en vanskelig regulerbar atferd, slik en finner det hos barn med opposisjonell atferdsforstyrrelse (ODD) og ved hyperaktivitet (ADHD). Metoden bygger på elementer fra tradisjonell leketerapi, sosial læringsteori, tilknytningsteori samt familieterapi. Barnets interaksjon med foreldrene søkes endret gjennom å styrke positive relasjoner mellom barn og foreldre. I praksis gjennomføres dette ved direkte veiledning til foreldrene gjennom øreplugg, mens foreldrene leker med barnet. Gjennomsnittlig behandlingstid for å oppnå mestringskriteriene er 15-20 timer, med store individuelle variasjoner.

#### Behandling av barn med atferdsvansker

Barn med symptomer på atferdsvansker utgjør 19,8 % (Bjørngaard, 2001) av de som henvises til poliklinikker i psykisk helsevern for barn og unge, og de utgjør en stor andel av de barna som er brukere av kommunale tiltak for barn som skolehelsetjeneste, PPT og barnevern. Når atferdsvanskene får utvikle seg gjennom ungdomstid og voksen alder, medfører det meget store omkostningene for det enkelte barn, familien og for samfunnet som helhet (Carr, 1999).

Dette har vært bakgrunnen for en stor satsing på forskning og utprøving av nye metoder med betydelig støtte fra Barne- og familiedepartementet. Først i et 5-årig ”Atferdsprosjekt”, som fra 2003 er over i en ny fase der videre utvikling og implementering av metoder over hele landet står i fokus. De metodene en har valgt å satse på i Norge er Webster-Strattons metode (Webster-Stratton & Hancock, 1998) som er et gruppebasert opplegg for den yngste aldersgruppen 4-8 år, Parent Management Training-Oregon (PMT-O) fra 5 til 12 år (Ogden, 1999) og Multisystemisk Terapi (MST) for barn og unge over 12 år (Hengeler et al., 1992).

Nært beslektet med disse metodene er Parent Child Interaction Therapy (PCIT), som hittil er en relativt ukjent metode i Norge. Både teoretisk og i form av å være manualisert, er den nært beslektet med de andre metodene som nå prøves ut. Den skiller seg ut fra PMT-O ved å bruke leken som virkemiddel i stedet for samtale, og det kan bety at for foreldrene til de minste barna kan PCIT være en mer egnet tilnærming enn PMT-O. PCIT skiller seg fra Webster-Strattons metode ved å være et tilbud for enkeltfamilier. En er dermed mindre avhengig av en stor pasienttilgang for å samle homogene behandlingsgrupper. Særlig på mindre steder vil individuelle tilnærminger være mer egnet som tilbud enn Webster-Strattons metode. Den individuelle tilnærmingen har dessuten klare fordeler når det gjelder muligheter for tilpasning av opplegget i vanlig klinisk bruk til det enkelte barn og familie. Individuell tilnærming kan derfor tenkes å gjøre det mulig å behandle vanskeligere saker der bildet preges av komorbidet og tilleggsvansker, slik at anvendelsesområdet for metoden blir vesentlig utvidet.

PCIT har de siste årene blitt tatt i bruk ved de fleste poliklinikker i psykisk helsevern for barn og unge i Sør- og Nord-Trøndelag. Gjennom dette arbeidet har en etablert en prosjektgruppe som har vært viktig i planleggingen av dette prosjektet. Prosjektgruppen har fortløpende evaluert erfaringene fra arbeidet med PCIT , og dette har fungert som en pilotstudie før dette prosjektet. Det er også etablert et samarbeid med sentrale personer fra forskningsmiljø i USA som vil kunne bistå prosjektet med opplæring og veiledning.

***Teorier/Forskning***

Det er i dag stor faglig enighet om at dysfunksjonell foreldre-barn interaksjon er sentralt i utvikling av atferdsvansker hos små barn (Campbell, 1990; Patterson,1982). Det har ført til utvikling av ulike former for intervensjon der disse mønstrene i samspillet mellom barn og foreldre søkes endret. Familie- og foreldretreningsintervensjoner er dokumentert å ha langt bedre effekt enn andre behandlingsformer når det gjelder evidensbasert behandling av atferdsvansker (McClelland og Werry, 2003).

Behan og Carr (2000) gjennomgikk i alt 24 undersøkelser av behandlingseffekt for atferdsvansker hos barn, og konkluderte med at atferdsorientert foreldretrening kombinert med trening av problemløsningsferdigheter for barna er særlig effektive for barn med diagnosen opposisjonell atferdsforstyrrelse (ODD) uten spesielle tilleggsvansker. ODD vil være den vanligste formen for atferdsvansker hos de minste barna, men atferdslidelse (Conduct Disorder – CD) utgjør hovedtyngden av atferdsvansker hos ungdom. ODD er en sterk risikofaktor for utvikling av CD.

Gallagher (2003) har gått gjennom alle undersøkelser av effekt av PCIT. Konklusjonen er en samlet støtte til PCIT som effektiv behandlingsform, og den anbefales som evidensbasert metode for behandling av yngre barn med atferdsvansker. Hun peker imidlertid på at mange av undersøkelsene er gjort i universitetsklinikker, hvor terapeutene har fått omfattende trening, og hvor undersøkelsen er ledd i hoved- eller doktorgradsarbeider. Det er dermed usikkert i hvilken grad resultatene kan generaliseres til en mer realistisk klinisk setting.

Så langt kategoriseres evidensen for PCIT som ”Probably efficatious treatments” (Hibbs, 2001). Når metoden så langt ikke er klassifisert som å ha en etablert dokumentert effekt, er det fordi kun en studie har brukt alternativ behandling som kontroll, de resterende undersøkelsene har basert seg på venteliste-kontroller. Denne effektstudien vil basere seg på alternativ behandling.

**Målsetting**

***Overordnet mål.***

Å vurdere effekt og nytte av PCIT i vanlige barne- og ungdomspsykiatriske poliklinikker i Norge.

#### Forskningsspørsmål

1. Reduseres symptomer på atferdsvansker og forekomsten av atferdslidelse hos barn i alderen 2-8 år i større grad hos barn og familier som mottar PCIT enn hos barn og familier som mottar standard behandling?
2. I hvilken grad vedlikeholdes denne effekten ved 6-måneders og 12 måneders oppfølging?
3. Kan denne effekten tilskrives endring i foreldrenes atferd overfor barna målt ved DPICS?
4. Medieres eller modereres behandlingseffekten av foreldrenes opplevde kontroll-lokus og depressive symptomer?
5. Vil metoden gi effekt når den brukes som intensiv behandling under 3 ukers familieinnleggelse?
6. Vil foreldres livskvalitet bedres når symptomer på atferdsvansker hos barnet reduseres?
7. Vil tilknytning mellom barn og foreldre endres dersom barnets atferdsvansker reduseres?
8. Vil en kunne observere / måle endring i familieklima dersom barnets atferdsvansker reduseres?

## Plan for gjennomføring

#### Deltakere

Barn i alderen 2-8 år ved inntak til prosjektet henvist til de BUP-Klinikkene i Trøndelag, som bruker PCIT i behandling av små barn med atferdsvansker, samt ved Klinikkavdeling for barn, Psykologisk institutt, NTNU.

#### Inklusjonskriterier

- Barnets alder mellom 2 og 8 år ved inntak
- Skåre >120 (norske normer) på ECBIs intensitetsskåre eller en nåværende diagnose innen spekteret disruptive behavioral disorder (ODD eller CD)
- Familien har gjennomført screeningprosedyre og undertegnet behandlingsavtale

***Eksklusjonskriterier***

- Psykose eller alvorlig rusmisbruk hos foreldre
- Mistanke om alvorlig mishandling
- Mental retardasjon hos barnet

# Prosedyre

Alle barn henvist for atferdsvansker eller hyperaktivitet/konsentrasjonsvansker screenes ved hjelp av ECBI (Eyberg & Pincus, 1999). Diagnoser settes for prosjektformål etter DSM-IV ved hjelp av K-SADS eller PAPA. Familiene undersøkes før behandlingsstart, ved behandlingsslutt, og 6 måneder etter behandlingsslutt. Da PCIT behandlingen vil variere i lengde etter hvor lang tid det tar å oppnå kriteriene i manualen, vil hver familie pares med sin kontrollfamilie, som blir undersøkt etter like lang tid som eksperimentfamilien. Familiene taes inn i prosjektet etter hvert som de kan tilbys PCIT eller standard behandling, slik at ventetid er like lang for begge gruppene. Dersom det oppstår ventetid til standard behandling, vil dette også bli praktisert balanserende i prosjektet.

Ved en del tilstander, i særlig grad ADHD og i noen grad ved ADD, vil medikamentell behandling være as inn i prosjektet slik at effekt av medisinering blir stabil gjennom prosjektperioden. Ut fra etiske hensyn kan stimulantia bli utprøvd/brukt i så vel kontroll- som eksperimentgruppe når det er indisert. Utprøving av medisineffekt forutsettes å være avsluttet før barnet tas inn i prosjektet slik at effekt av medisinering blir stabil gjennom prosjektperioden.

Fremgangsmåten i behandlingsmetoden er detaljert beskrevet av Hembree-Kigin og McNeil (1995). Det er utarbeidet manual for metoden, denne er oversatt og tilpasset norske forhold av universitetslektor / psykologspesialist Anne Kristine Wormdal (2005). Behandlere ved de ulike klinikkene har fått opplæring i bruk av manualen, og behandlingen vil gjennomføres i henhold til denne.

# Design

RCT studie. Eksperimentgruppe (n=35) gis PCIT. Kontrollgruppe (n=35) gis ordinær behandling ved de gjeldende poliklinikkene. Denne behandlingen vil variere mellom behandlere og mellom klinikker, og kan omfatte foreldreveiledning, familieterapi, veiledning til barnehage eller skole, leketerapi, kognitiv atferdsterapi, og / eller medikamentell behandling særlig med stimulantia ved hyperkinesi.

Klinikker der Webster-Stratton behandling eller PMT-O tilbys som standard behandling, inkluderes ikke i undersøkelsen.

Randomisering skjer ved tilfeldig nummertrekking (SPSS).

**Prosjektplan**

Følgende BUP - klinikker er inkludert i prosjektet, og anslås i løpet av den 2-årige prosjektperioden å bidra med angitt antall saker til undersøkelsen:

| **Klinikk** | **PCIT-terapeut** | **I opplæring** | **PCIT-gruppe** | **Kontrollgruppe** |
| --- | --- | --- | --- | --- |
| Røros | 1 | 1 | 5 | 5 |
| Orkdal | 3 |  | 10 | 10 |
| Fosen | 1 | 1 | 5 | 5 |
| Levanger | 2 |  | 10 | 10 |
| Namsos | 1 | 1-2 | 5 | 5 |

Etter hvert synes det tekniske utstyret å fungere godt for alle enhetene, og klinikker hvor bare en behandler har anvendt metoden, vil i løpet av året få minst en medarbeider med seg. Metoden er kjent i de aktuelle klinikkene, og er anerkjent som egnet metode for pasientgruppen.

##### Organisering/Arbeidsform og kritiske faktorer

Behandlernes kompetanse og kapasitet er kritiske faktorer i gjennomføringen av prosjektet. Selv om metoden er manualisert, så er det svært langt fra å kjenne oppskriften til å gjennomføre behandlingen med tilstrekkelig kvalitet. Behandlerne som skal delta har fullført opplæring, men har ulik grad av trening. Det vil bli nødvendig å lære opp nye behandlere, noe som vil kreve kontinuerlig opplæring og veiledning av behandlere. Terapeutenes anvendelse av manualen og gjennomføring av terapiforløpene vil periodevis bli evaluert, likeledes vil kommunikasjonskodernes intra-rater reliabilitet bli sjekket i henhold til gjeldende standarder (Eyberg et al., 1994).

# Statistisk styrke

Effektstørrelsene ved PCIT synes lik den som er oppnådd ved Webster-Stratton metodikk. Antall deltagere vil derfor basere seg på gruppestørrelser som er gjengs i tidligere studier. ECBI er et kontinuerlig mål, noe som vil øke sensitiviteten for å oppdage effektulikheter mellom eksperiment- og kontrollgruppe. Da spredningen antas lik i både eksperiment og kontrollgruppe, settes antall deltagere til 35 i begge grupper. Med foreliggende pasienttilgang og en forventet deltagelsesprosent på 70, estimeres tiltaksfasen å strekke seg over to år.

# Blindhet

ECBI og K-SADS / PAPA taes initialt før allokering. Familiekommunikasjonen taes opp på video for koding. Ved BUP - Sør-Trøndelag finnes kodere, som har sagt seg villige til å kode materialet (Eyberg et al,1994). Denne klinikken inngår ikke i undersøkelsen og kodere vil således være blinde. K-SADS taes av medarbeidere som er ukjent med hvilket tilbud som er gitt familiene.

##### Måleinstrumenter

#### Kartlegging av barnet

**Anamnestiske opplysninger**. Eget skjema utarbeidet, basert på klinikkenes ordinære praksis.

**Aschenbach**. Child Behavior Check List (CBCL) og Teacher Rating Form (TRF) vil bli brukt ettersom det er innført som standard registrering av barnets symptomer før og etter behandling i poliklinikkene. (Achenbach, 1992).

**Kiddie-SADS-PL**. Semistrukturert foreldreintervju for kartlegging av psykiatriske symptomer for barn i aldersgruppen 6-18 år. Oversatt ved R-BUP/ NTNU, og i bruk ved poliklinikkene i Trøndelag. Intervjuet er brukt i en større epidemiologisk studie i samme distrikt (Sund, 2003).

**The Preschool Age Psychiatric Assessment (PAPA)**. Et strukturert foreldreintervju for å diagnostisere psykiatriske lidelser for barn i førskolealder. Oversetting og opplæring av intervjuere/skårere gjennomføres i sammenheng med undersøkelse av 4-åringer i Trondheim kommune (Berg- Nilsen og Wichstrøm). Denne opplæringen planlegges gjennomført høsten 2006, og barn i denne aldersgruppen vil først inkluderes i prosjektet når PAPA kan tas i bruk.

**Eyberg Child Behavior Inventory (ECBI).** Et anbefalt og mye brukt registreringsskjema for atferdsvansker hos små barn. Gjennom Webster-Stratton prosjektet standardisert i Norge.

**Working Model of the Child Interview (WMCI**) (Rosenblum, K.L., Zeanah, C., McDonough, S. & Muzik, M., 2004). Foreldreintervju, som vil gi en beskrivelse av barnets tilknytningsmønster i relasjon til den av foreldrene som intervjues.

# *Kartlegging av foreldre-barn interaksjonen*

**Dyadic Parent-Child Interaction Coding System (DPICS).** System for skåring av foreldre-barn interaksjon basert på videoopptak eller observasjon. Det vil tas et videoopptak av samspillet før og etter behandling. I tillegg vil hver enkelt behandlingstime starte med en 5-minutters observasjon av samspillet, som skåres "live".

# *Kartlegging av foreldrefungering*

**Beck Depression Inventory (BDI).** Det er ofte sammenheng mellom depresjon hos foreldre og atferdsvansker hos barn. Årsakene kan både være at deprimerte foreldre ikke gir barna tilstrekkelig omsorg / oppmerksomhet, og at det å ha et atferdsvanskelig barn kan gi en sterk følelse av å være mislykket som foreldre. Hood & Eyberg (2003) fant imidlertid ikke signifikante endringer i BDI fra før til etter behandling

**Parental Locus of Control – Short Form (PLOC-SF).** Spørreskjema til foreldre, måler foreldrenes opplevelse av å mestre barnets atferd. PLOC-SF har vist seg å være relativt sensitivt til effekt av behandling (Campis, Lyman & Prentice-Dunn, 1986).

#### Kartlegging av familieforhold

**Family Assessment Measure (FACES-IV).**

##### Behandlingsprosedyre for PCIT

Fremgangsmåten i behandlingsmetoden er detaljert i beskrevet av manual utarbeidet av McNeil i samarbeid med Eyberg (2004?). Manualen er oversatt og tilpasset norske forhold av Wormdal (2005).

***Forberedelse og utredning.***

Før behandlingen starter gjennomføres en inntaksvurdering med samtale med foreldrene om barnets og familiens vansker. Hensikten med dette er å finne fram til gode felles mål for behandlingen. Et viktig prinsipp er at målsettingen for behandlingen skal være relativt vid, og ikke begrenset til en bestemt målatferd.

***Trinn1: Foreldrene lar barnet lede interaksjonen***

Første steg er en time med foreldrene alene der den første del av behandlingen gjennomgås.

I de følgende timene kommet barnet sammen med foreldrene. Hver time starter med en kort samtale med fokus på hvordan det har gått siden sist. Deretter går terapeuten inn i observasjonsrommet, mens barnet og den ene av foreldrene leker sammen. Først gjennomføres en registreringsperiode på 5 minutter der foreldrenes atferd skåres med DPICS. Deretter fortsetter leken, mens mor eller far får veiledning fra terapeuten på øret.

Foreldrene hjelpes til å forsterke barnets positive atferd, samtidig som de lærer å overse barnets negative atferd. De veiledes i å følge barnet ved å beskrive det barnet gjør i leken, svare på alle verbale initiativ fra barnet, rose barnets prososiale atferd, samt å gi barnet ulike former for nonverbal støtte og bekreftelse. Samtidig veiledes de i å redusere antall beskjeder, spørsmål, kritikk og andre negative reaksjoner. Gjennom rollespill får foreldrene prøvd ut og vent seg til arbeidsformen og det tekniske utstyret. Behandlingen forutsetter i tillegg en 5 minutters daglig treningsøkt hjemme for hver av foreldrene.

Veiledningen gis i form av korte, presise kommentarer for å ikke forstyrre flyten i samhandlingen mellom foreldre og barn. I startfasen bør hvert utsagn fra foreldrene utløse en kommentar fra terapeuten, som så gjennom terapiforløpet gradvis blir mindre aktiv. Terapeuten kan også komme med forslag til hva foreldrene skal si, gi vennlige korreksjoner, eller påpeke og forklare sammenhenger i interaksjonen med barnet. Timen avsluttes med en ny kort samtale mellom terapeuten og foreldrene. Fremgang målt i registreringstiden fremheves, og neste times utfordring omtales.

Før foreldrene kan gå videre til andre del må mestringskriteriene være oppnådd. Gjennom overlæring vil en sikre at ferdighetene automatiseres og generaliseres til hverdagen hjemme. Det stilles ikke noe krav til at begge trinn av behandlingen skal gjennomføres, ofte vil en etter den første delen se at både interaksjonen og barnet har forandret seg så mye at terapien kan avsluttes. Om en går videre, avhenger av om de mål en har satt seg for terapien er oppnådd.

***Trinn 2: Foreldrene leder interaksjon***

Hovedhensikten med denne delen av behandlingen er å redusere barnets problematferd. En starter igjen med en opplæringstime for foreldrene, og fortsetter med direkte trening på ulike former for grensesetting. Formidling av tydelige og aldersadekvate beskjeder til barnet er avgjørende, og viktigheten av forutsigbarhet, konsistens og utholdenhet i gjennomføring av grensesetting er sentrale tema. Barnet informeres om forventet/ønsket atferd, samt om konsekvenser (tenkepause) dersom det ikke samarbeider. Rollespill og dukker brukes gjerne når en viser barnet hvordan det nå skal trene seg på å høre etter foreldrene. Trening på avspenning, ulike måter å roe seg ned på og pusteteknikk introduseres for foreldrene som teknikker for å mestre dette. Treningen går ofte gjennom flere steg fra ”høre-etter-øvelser” til trening i virkelige situasjoner, etablering av husregler, trening med søsken til stede, osv.

**Tidsplan**

Prosjektet er planlagt å starte fra 01.03.06, og skal gå fram til 31.02.08. Rekruttering av familier vil pågå de to første årene. Data opp til behandlingsslutt vil inngå i avhandlingen. For å fremskynde rapporteringen vil prøveanalyser av materialet kjøres og utskriving begynne før de siste data ved behandlingsslutt er samlet inn.

**Prosjektets nytteverdi og vitenskaplig betydning**

Som det er vist til tidligere, vil det både samfunnsmessig og for den enkelte ha stor verdi å kunne gi tidlig og god behandling for barn med atferds- og samspillsvansker. Dersom en gjennom dette prosjektet kan avklare om PCIT har god effekt ved behandling i vanlige klinikker, så vil enda en metode være tilgjengelig for bruk i hjelpeapparatet. I tillegg vil PCIT ved dette internasjonalt kunne gis en etablert status som dokumentert effektiv metode. PCIT har en form og organisering som gjør den enkel å implementere i små og mellomstore poliklinikker. Bruk av PCIT kan derfor gjøre tilbudet om effektiv hjelp mer tilgjengelig over hele landet, og den kan supplere tilbudet som allerede eksisterer med PMT og Webster-Stratton-grupper.

### Etiske betraktninger

Foreldrene vil få skriftlig og muntlig orientering om prosjektet med forespørsel om å delta. Skriftlig samtykke vil bli innhentet fra foreldrene.

Gjennom erfaringene fra de foreløpige utprøvingene vet en at PCIT i starten kan oppleves som noe krevende for foreldrene fordi de må eksponere seg og samspillet sitt med barnet for andre. Dette er som regel noe som går over når de har etablert tillit til behandleren. Behandlingen oppleves derimot nesten utelukkende positivt av barna som er med, fordi det gir dem gode opplevelser med mye positiv oppmerksomhet fra foreldrene. Treningen på grensesetting kan kanskje være mer krevende for barnet, men sannsynligvis vil de konfliktene som kan oppstå i terapirommet være mildere og mer kontrollert enn det de til daglig opplever hjemme.

Annen dokumentert effektiv behandling, i særdeleshet stimulantia ved hyperaktivitet vil kunne bli gitt til begge grupper når det er indisert, og slik følge vanlig praksis. Det samme gjelder rådgiving og informasjon til barnehage og skole når det er aktuelt.

**Publisering/Rapportering**

Resultater fra prosjektet vil bli beskrevet i artikkelform for publisering i et omfang som gjør at det vil fylle kravene i forbindelse med en Ph.d.-grad, dvs. at det taes sikte på fire internasjonale publikasjoner i internasjonale tidsskrift med fagfelle vurdering. På lengre sikt vil imidlertid prosjektet gi data til flere artikler.

**Budsjett**

**Kostnader som dekkes av andre**

I kostnadsoverslaget er det forutsatt at poliklinikkene som deltar dekker det vesentlige av driftskostnadene ved prosjektet som tid og utgifter til reise for opplæring/veiledning for behandlerne, lønnsutgifter til behandlere, samt opptrykk av materiell, videoutstyr og porto. Lønn til undersøkelser som normalt ikke vil inngå i klinikkenes ordinære tilbud til pasientene slik som lønn til studenter for ekstra arbeid som terapeuter, koding av DPICS og gjennomføring av K-SADS-PL ved oppfølging søkes finansiert fra annet hold.

**Referanser**

Achenbach, T. M. (1992). Manual for the Child Behaviour Check List/2-3 and 1992 profile.

Burlington: University of Vermont, Department of Psychiatry.

Behan, J. & Carr, A. (2000) Oppositional defiant disorder. I: Carr,A. (Ed.) *What works with Children and adolescents.* London: Routledge.

Bjørngaard, J.H. (2001) *Samdata Psykisk helsevern*. Tabeller. SINTEF Unimed, Rapport 2001.

Bjørseth, Å. & Wormdal, A.K. (2004) Parent Child Interaction Thearpy (PCIT) – Presentasjon av en metode. *Upublisert manuskript.*

Brestan, E., Eyberg, S., Boggs, S., & Algina, J. (1997) Parent Child Interaction therapy. Parent perceptions of untreated siblings. Child and Family Behavior Therapy, 19, 13-28.

Campis, L.K., Lyman, R.D. & Prentice-Dunn, S. (1986) The Parental Locus of Control Scale: Development and Validation. Journal of Clinical Child Psychology, Vol.15, 1986.

Campbell, S.B. (1990) *Behavior problems in preschool children: Clinical and developmental issues*. New York: Guilford Press.

Carr, A. (1999) *The handbook of child and adolescent clinical psychology. A contextual approach.* London: Routledge.

Eisenstadt, T.H., Eyberg, S., McNeil, C.B., Newcomb, K., & Funderburk, B. (1993) Parent-child interaction therapy with behaviour problem children: Relative effectiveness of two stages and overall treatment outcome. *Journal of Clinical Child Psychology*, 22. 42-51.

Eyberg, S., Boggs, S., & Algina, J. (1995) Parent-child interaction therapy. A psychosocial model for treatment of young children with conduct problem behavior and their families. Psychopharmacology Bulletin, 31, 83-91.

Eyberg, S. & Pincus, D. (1999) Eyberg Child Behavior Inventory and Sutter-Eyberg Student Behavior Inventory: Professional manual. Odessa,FL: Psycholocical Assessment Resources.

Eyberg, S.M, Bessmer, J., Newcomb, K., Edwards, D. & Robinson, E.A. (1994). Dyadic parent-child interaction coding system. A manual. Upublisert manuskript. University of Florida: http://www.pcit.org

Eyberg, S.M. & Boggs, S.R.(1998). Parent-child Interaction therapy: A psychosocial intervention for the treatment of young conduct-disordered children. I Briesmeister, J.M. and Schaefer, C.E. (Eds.) Handbook of parent training: parents as co-therapists for children`s behavior problems. New York: Wiley.

Eyberg, S.M., (1998) Parent-Child Interaction Therapy. Intergration of traditional and behavioral concerns. *Child and Family Behavior Therapy,10*, 33-46.

Gallagher, N. (2003) Effects of Parent-Child Interaction Therapy on Young Children with Disruptive Behavior Disorders. *Bridges*, Vol.1, No. 4.

Hembree-Kigin, T. & McNeil, C.B. (1995). *Parent–Child Interaction Therapy.* New York: Plenum.

Henggeler, S. W., Melton, G. B., & Smith, L. A. (1992). Family preservation using multisystemic therapy: an effective alternative to incarcerating serious juvenile offenders. Jo*urnal of the American Academy of Child and Adolescent Psychiatry, 41*, 868 – 874.

Hood, K. K. & Eyberg, S. M.(2003). Outcomes of Parent-Child Interaction Therapy: Mothers’ Reports of Maintenance Three to Six Years after Treatment. Journal of Clinical Child and Adolescent Psychology, 2003, Vol. 32, No. 3.

Hibbs, E.D. (2001) Evaluating empirically based psychotherapy research for children and adolescents. European Child & Adolescent Psychiatry, 10, 2001.

McClellan, J. M. & Werry, J.S. (2003) Evidence-Based Treatments in Child and Adolescent Psychiatry: An inventory. Journal of the American Academy of Child & adolescent Psychiatry, Vol. 42(12)

McNeil, C. B. et al. (1991) Parent-Child Interaction Therapy with behavior problem children: Generalization of treatments effects to the school setting. Journal of Clinical Child Psychology 20: 140-151.

Ogden, T. (1999). Antisosial atferd og barneoppdragelse. ”Parent Management Training” som foreldreopplæring. Spesialpedagogikk, 6, 1999.

Patterson, G.R. (1982) *Coercive family process*. Eugene, OR: Castalia.

Rosenblum, K.L., Zeanah, C., McDonough, S. & Muzik, M. (2004). Video-taped coding of

working model of the child interviews: a viable and useful alternative to verbatim transcripts?

*Infant Behavior & Development*, *27*, 544–549.

Webster-Stratton, C. & Hancock, L. (1998). Training for parents of young children with conduct problems: content, methods, and therapeutic processes. I Briesmeister,

J. M. & Schaefer, C. E. (Eds.) Handbook of parent training: parents as co-therapists for children`s behavior problems (ss. 98-152). New York: Wiley.
